# Local microvascular leakage promotes trafficking of activated neutrophils to remote organs

**DOI:** 10.1172/JCI133661

**Published:** 2020-03-23

**Authors:** Charlotte Owen-Woods, Régis Joulia, Anna Barkaway, Loïc Rolas, Bin Ma, Astrid Fee Nottebaum, Kenton P. Arkill, Monja Stein, Tamara Girbl, Matthew Golding, David O. Bates, Dietmar Vestweber, Mathieu-Benoit Voisin, Sussan Nourshargh

**Affiliations:** 1 William Harvey Research Institute, Barts and The London School of Medicine and Dentistry, Queen Mary University of London, London, United Kingdom.; 2 Department of Vascular Cell Biology, Max Planck Institute for Molecular Biomedicine, Münster, Germany.; 3 Division of Cancer and Stem Cells, School of Medicine, Queen’s Medical Centre, University of Nottingham, Nottingham, United Kingdom.; 4 Centre for Inflammation and Therapeutic Innovation, Queen Mary University of London, London, United Kingdom.

**Keywords:** Inflammation, Vascular Biology, Chemokines, Neutrophils, endothelial cells

## Abstract

Increased microvascular permeability to plasma proteins and neutrophil emigration are hallmarks of innate immunity and key features of numerous inflammatory disorders. Although neutrophils can promote microvascular leakage, the impact of vascular permeability on neutrophil trafficking is unknown. Here, through the application of confocal intravital microscopy, we report that vascular permeability–enhancing stimuli caused a significant frequency of neutrophil reverse transendothelial cell migration (rTEM). Furthermore, mice with a selective defect in microvascular permeability enhancement (*VEC-Y685F-ki*) showed reduced incidence of neutrophil rTEM. Mechanistically, elevated vascular leakage promoted movement of interstitial chemokines into the bloodstream, a response that supported abluminal-to-luminal neutrophil TEM. Through development of an in vivo cell labeling method we provide direct evidence for the systemic dissemination of rTEM neutrophils, and showed them to exhibit an activated phenotype and be capable of trafficking to the lungs where their presence was aligned with regions of vascular injury. Collectively, we demonstrate that increased microvascular leakage reverses the localization of directional cues across venular walls, thus causing neutrophils engaged in diapedesis to reenter the systemic circulation. This cascade of events offers a mechanism to explain how local tissue inflammation and vascular permeability can induce downstream pathological effects in remote organs, most notably in the lungs.

## Graphical abstract

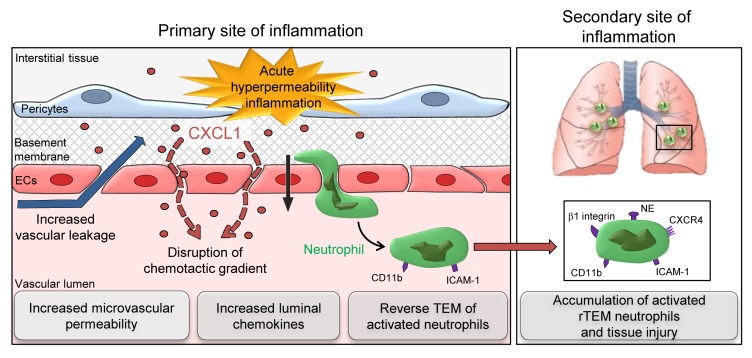

## Introduction

Acute inflammation is a critically important pathophysiological response to a local stimulus (e.g., bacterial infection) characterized by local tissue infiltration of neutrophils and tissue swelling (edema). These responses typically begin within minutes after a stimulus and collectively support the activation of essential immunoregulatory, proinflammatory, and proresolution pathways required for effective host defense and tissue repair. Excessive and/or inappropriately triggered neutrophil migration and increased vascular leakage can also be the underlying cause of a vast range of acute and chronic inflammatory disorders, such as acute lung injury and rheumatoid arthritis, and are as such well-established antiinflammatory therapeutic targets (1, 2). Central to regulation of these responses are endothelial cells (ECs) that line the inner aspect of all blood vessels and provide the principal barrier to migrating immune cells and blood-borne macromolecules. With respect to neutrophil trafficking, ECs provide critical proadhesive and other effector molecules that facilitate a cascade of neutrophil-EC interactions, such as neutrophil rolling, firm arrest, and luminal crawling. These events that are generally considered to be prerequisite to breaching of the endothelium (3). Transendothelial cell migration (TEM) is supported by an array of EC junctional molecules, most notably PECAM-1 (CD31), members of the junctional adhesion molecule (JAM) family, VE-cadherin, and others (3–5). In addition, we recently demonstrated the importance of retaining directional cues within EC junctions in facilitating luminal-to-abluminal neutrophil breaching of the endothelium (6).

With respect to vascular leakage, under basal conditions the microvascular endothelium has a low permeability to plasma proteins, and as such establishes an oncotic gradient that opposes the vascular hydrostatic pressure that would otherwise excessively move water and solutes into the tissue. This situation rapidly changes in inflammation, enabling immediate supply of plasma proteins (e.g., complement components and Abs) to injured or infected organs through increased permeability of the vascular endothelium (7). Leakage of macromolecules in inflammation largely occurs via a paracellular route involving loosening of EC junctions (5, 7, 8) mediated via actinomyosin-based contraction of ECs and destabilization of junctional contacts. The latter most notably involves downregulation of the adhesive functions of VE-cadherin (5, 8), with inflammation-induced increased vascular permeability being associated with elevated tyrosine phosphorylation of components of the VE-cadherin–catenin complex (5, 9). Of particular significance, investigations using knockin mice expressing specific point mutations in VE-cadherin tyrosine residues (i.e., VEC-Y685F and VEC-Y713F) have categorically identified distinct molecular mechanisms in governing the passage of neutrophils and macromolecules through EC junctions (9). These findings are in line with early seminal works that definitively uncoupled in a temporal and spatial manner leukocyte extravasation from increased vascular permeability (10, 11).

Despite this, the close association of stimulated neutrophil transmigration and vascular permeability has historically attracted much attention toward their potential interplay, and indeed, it is now generally accepted that neutrophils can promote microvascular leakage in the early stages of an acute inflammatory response (12, 13). However, whether vascular permeability can regulate neutrophil migration remains poorly investigated and is contentious. To address this fundamental element of acute inflammation we used confocal intravital microscopy (IVM) to simultaneously analyze vascular leakage and neutrophil trafficking in inflamed tissues. Increased microvascular leakage did not influence the overall magnitude of neutrophil infiltration into tissues over several hours. Surprisingly, however, leaky microvessels promoted a rapid and significant frequency of neutrophil reverse TEM (rTEM) (~20%–40%) in the early phases of hyperpermeability reactions. This aberrant behavior was caused by an immediate increase in diffusion of interstitial chemokines into the vascular lumen, a response that disrupted the correct localization of chemotactic cues within the venular wall niche. Furthermore, through development of an in vivo cell labeling method, we provide direct evidence for the ability of rTEM neutrophils that have stemmed from an inflammatory vascular leakage site to traffic to the lungs. Functionally, rTEM neutrophils were activated, and their presence within the pulmonary vasculature was aligned with sites of vascular injury, suggesting a pathological role for this subset of neutrophils. Collectively, by finding that local microvascular leakage induction facilitates movement of interstitial chemokines through EC junctions and entry into the vascular lumen, we have discovered an additional mechanism for promotion of neutrophil rTEM in vivo. Importantly, our findings directly link this cascade of events with the capacity of local hyperpermeability reactions to elicit remote organ damage.

## Results

### Leaky venules support neutrophil rTEM.

**Figure 1:**
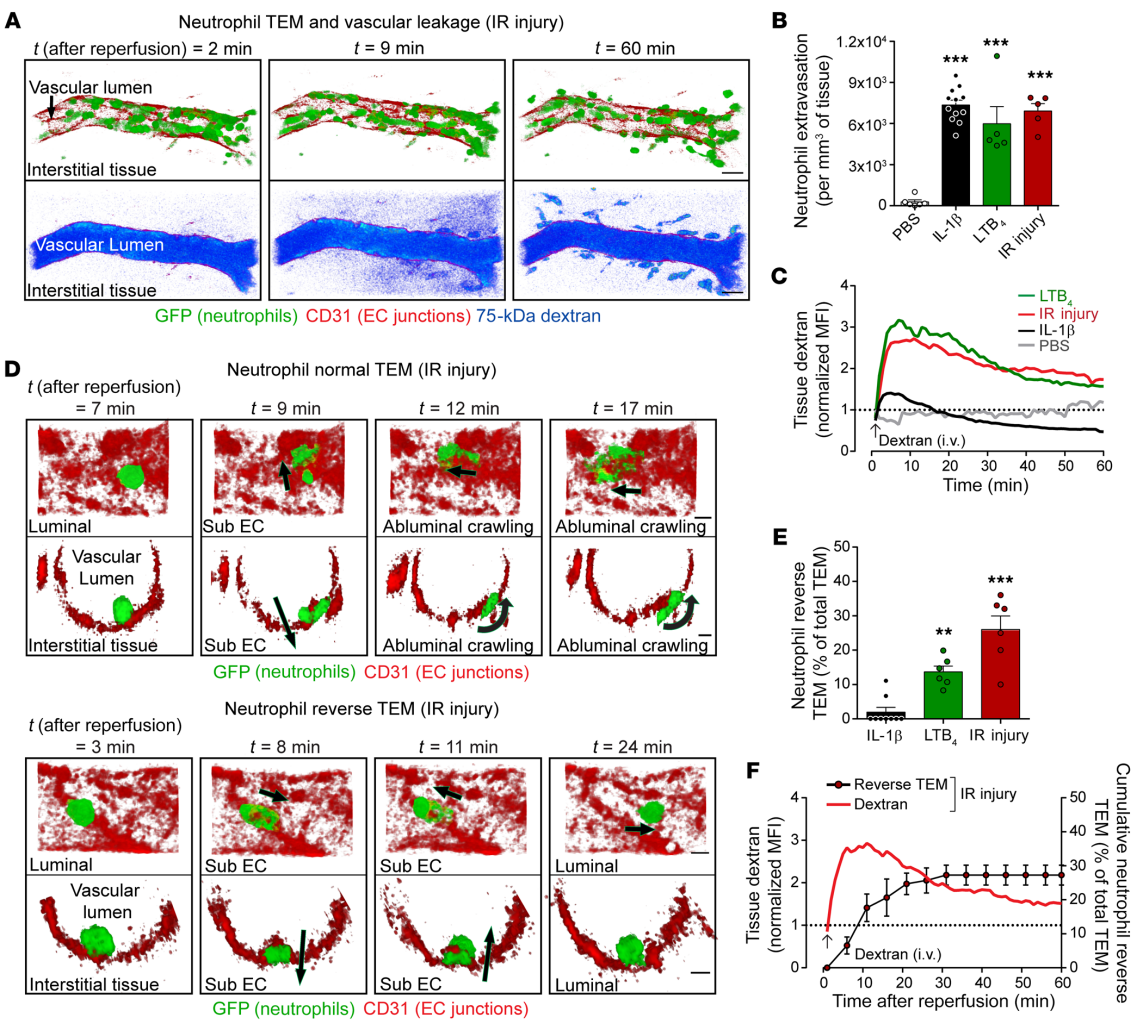
Hyperpermeability inflammatory reactions are associated with neutrophil reverse transendothelial migration. Cremaster muscles of *LysM-EGFP-ki* mice were subjected to IL-1β– or LTB_4_-induced inflammation (120 and 30 minutes, respectively), or to IR injury (40 minutes ischemia + 1–2 hours reperfusion) and analyzed by confocal IVM. PBS-treated or sham-operated animals were used as control. AF647-labeled anti-CD31 mAb was injected i.s. to visualize EC junctions (red) and i.v. fluorescent (75 kDa) TRITC-dextran was used to visualize vascular leakage (blue pseudocolor intensity). (**A**) Representative images of IR-stimulated cremasteric venules (see Supplemental Video 1), showing the development of an inflammatory response in terms of neutrophil migration (green GFP^bright^ neutrophils; top panels) and dextran leakage (blue; bottom panels) at the indicated times after reperfusion. Scale bars: 20 μm. (**B**) Total neutrophil extravasation (*n* = 5–12 mice/group). (**C**) Time course of dextran accumulation in the perivascular region of a selected postcapillary venule (*n* = 3–10 mice/group). (**D**) Representative images of an IR-stimulated cremasteric postcapillary venule at different times after reperfusion (see Supplemental Videos 2 and 3) illustrating a normal neutrophil TEM (top) and a reverse TEM (bottom) event. Luminal and cross-sectional views with arrows indicating the direction of motility of the indicated neutrophil. Scale bars: 5 μm. (**E**) Frequency of neutrophil reverse TEM events in relation to total TEM events of 20.7 ± 2.1 (IL-1β), 31.3 ± 5.9 (LTB_4_), and 15 ± 2.4 (IR injury) per 300-μm venular segment within 2-hour microscopy periods (mean ± SEM, *n* = 6–9 mice/group). (**F**) Temporal association of dextran leakage and cumulative frequency of neutrophil reverse TEM (*n* = 4 mice). Data are mean ± SEM (each symbol represents 1 mouse/independent experiment). Statistically significant differences from PBS-treated (**B**) or IL-1β–treated (**E**) mice are indicated by **P < 0.01; ***P < 0.001, 1-way ANOVA followed by Bonferroni’s post hoc test.

To directly investigate potential associations between neutrophil trafficking and microvascular permeability, inflamed mouse cremaster muscles were analyzed for neutrophil breaching of EC junctions and microvascular leakage in real time and in 3D (i.e., 4D) by confocal IVM. Briefly, the model employs *LysM-EGFP-ki* mice in conjunction with in vivo labeling of EC junctions using locally applied, nonblocking Alexa Fluor 647–labeled (AF647-labeled) anti-CD31 mAb (14). Neutrophil TEM was analyzed through tracking of GFP^bright^ neutrophils, and microvascular leakage was quantified by measuring interstitial accumulation of intravenously injected plasma protein tracer TRITC-dextran (MW ~75 kDa) (Figure 1A and Supplemental Video 1; supplemental material available online with this article; https://doi.org/10.1172/JCI133661DS1). In initial studies we analyzed inflammatory responses induced by local injections of leukotriene B_4_ (LTB_4_) and IL-1β and by a local pathophysiological insult of ischemia/reperfusion (IR) injury (all within 2 to 4 hours). Although these acute reactions elicited significant and comparable levels of total neutrophil infiltration into tissues within the overall test periods, as compared with control responses (Figure 1B), marked and rapid increased vascular leakage was only detected in tissues stimulated with LTB_4_ and IR (Figure 1C). Of note, over time, hyperpermeability reactions were associated with uptake of tissue-infiltrated dextran by perivascular cells (Figure 1A) but these regions were excluded from the image analysis process.

Most importantly, in contrast to the normal neutrophil TEM observed in IL-1β–stimulated tissues (i.e., neutrophils breaching EC junctions in a luminal-to-abluminal direction and without pause; Figure 1D and Supplemental Video 2), inflammatory reactions that increased vascular leakage were associated with the occurrence of neutrophil rTEM (Supplemental Video 3). Specifically, in LTB_4_- and IR-stimulated tissues, and in line with our previous reports (14, 15), a significant proportion of neutrophils that initiated TEM by extending protrusions through EC junctions rapidly retracted their cell body, exhibited reverse motility through EC junctions, and ultimately returned to the blood circulation (Figure 1D). This mode of neutrophil TEM accounted for approximately 20%–40% of all TEM events driven by LTB_4_ and IR but was rarely seen in IL-1β–stimulated tissues (<5%) (Figure 1E). Using the IR reaction to analyze the onset of this phenomenon, we noted that approximately 62% of the rTEM events occurred within the first 10 minutes of the reperfusion phase (Figure 1F). In contrast, normal neutrophil TEM, as indicated via neutrophil accumulation in the perivascular tissue, was more sustained and continued to develop over the 2-hour reperfusion period analyzed (data not shown). Of significance, although a close temporal association was observed between dextran leakage and frequency of neutrophil rTEM, the former exhibited an earlier onset (i.e., within less than 5 minutes after reperfusion; Figure 1F). This raised the possibility that increased EC permeability can influence the directionality of neutrophil TEM, prompting us to investigate the impact of vascular leakage–enhancing stimuli, histamine and VEGF-A_164_ (VEGF), on rTEM.

**Figure 2:**
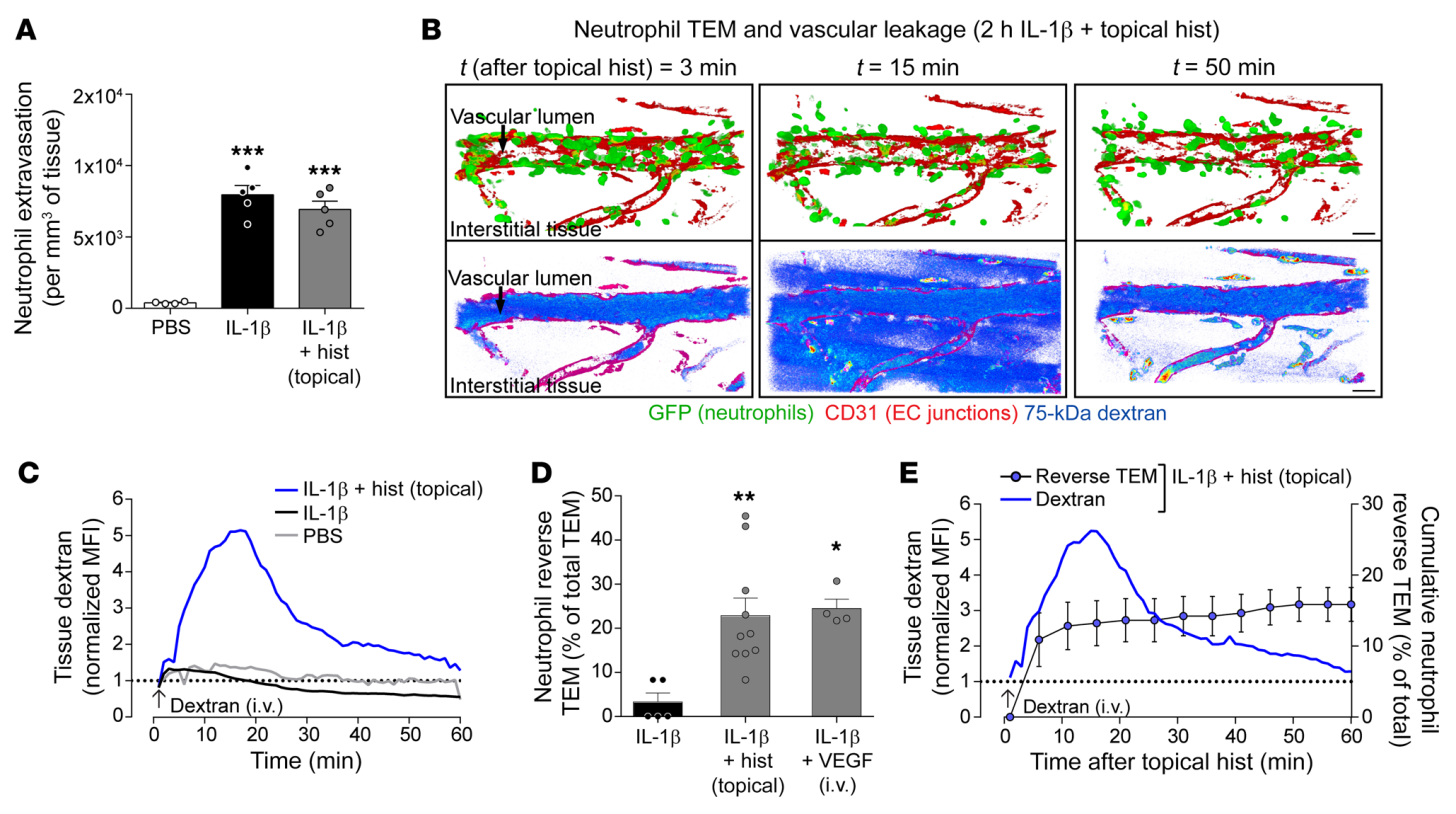
Induction of microvascular leakage promotes neutrophil reverse transendothelial migration. Cremaster muscles of *LysM-EGFP-ki* mice were subjected to IL-1β–induced inflammation for 120 minutes and analyzed by confocal IVM. AF647-labeled anti-CD31 mAb (i.s.) and 75-kDa TRITC-dextran (i.v.) were used to visualize EC junctions (red) and vascular leakage (blue pseudocolor intensity), respectively. Mice were superfused with histamine (30 μM) or injected i.v. with VEGF (4 μg) 2 hours after IL-1β stimulation. (**A**) Total neutrophil extravasation (*n* = 4–5 mice/group). (**B**) Representative images of a postcapillary venular segment subjected to IL-1β plus histamine stimulation at different time points after application of histamine, illustrating neutrophil TEM and dextran leakage responses (see Supplemental Video 4). Scale bars: 20 μm. (**C**) Time course of dextran accumulation in the perivascular region of a selected postcapillary venule (*n* = 3–9 mice/group). (**D**) Frequency of neutrophil reverse TEM events in relation to total TEM events of 15.2 ± 2.4 (IL-1β), 19.8 ± 2.9 (IL-1β + hist), and 21 ± 3.6 (IL-1β + VEGF) per 300-μm venular segment within 2-hour microscopy periods (mean ± SEM, *n* = 4–10 mice/group). (**E**) Temporal association of dextran leakage and cumulative frequency of neutrophil reverse TEM (*n* = 7–9 mice/group). Data are represented as mean ± SEM (each symbol represents 1 mouse/independent experiment). Statistically significant differences from PBS-treated (**A**) or IL-1β–treated (**D**) mice are indicated by *P < 0.05; **P < 0.01; ***P < 0.001, 1-way ANOVA followed by Bonferroni’s post hoc test.

In agreement with previously published reports (16, 17), topical application of histamine to exteriorized cremaster muscles promoted neutrophil rolling but failed to induce neutrophil firm arrest or transmigration (Supplemental Figure 1, A–C). Similarly, topical application of histamine to exteriorized IL-1β–stimulated cremaster muscles caused no significant increase in neutrophil adhesion or transmigration beyond that seen with IL-1β alone (Supplemental Figure 1D and Figure 2A). As anticipated, topical histamine induced a marked vascular leakage response in IL-1β–treated tissues as compared with tissues treated with just IL-1β or PBS (Figure 2, B and C, and Supplemental Video 4); however, most intriguingly, it promoted a significant frequency of neutrophil rTEM events (~20%; Figure 2D and Supplemental Video 4). Similarly, although i.v. VEGF induced a notable vascular leakage response in mice treated with local IL-1β (Supplemental Figure 1E) without affecting total neutrophil infiltration (Supplemental Figure 1F), it caused a dramatic increase in frequency of neutrophil rTEM (~25%; Figure 2D). Furthermore, in animals treated with IL-1β plus histamine, maximal neutrophil rTEM was again detected rapidly after topical application of histamine (~80% within 20 minutes), in line with the rapid stimulated vascular leakage (Figure 2E). Collectively, the present findings demonstrate a previously unappreciated link between induction of vascular leakage and aberrant neutrophil TEM, with the induction of leakage emphatically affecting the directional migration of neutrophils through EC junctions.

### Genetic defect in microvascular leakage suppresses neutrophil rTEM.

**Figure 3:**
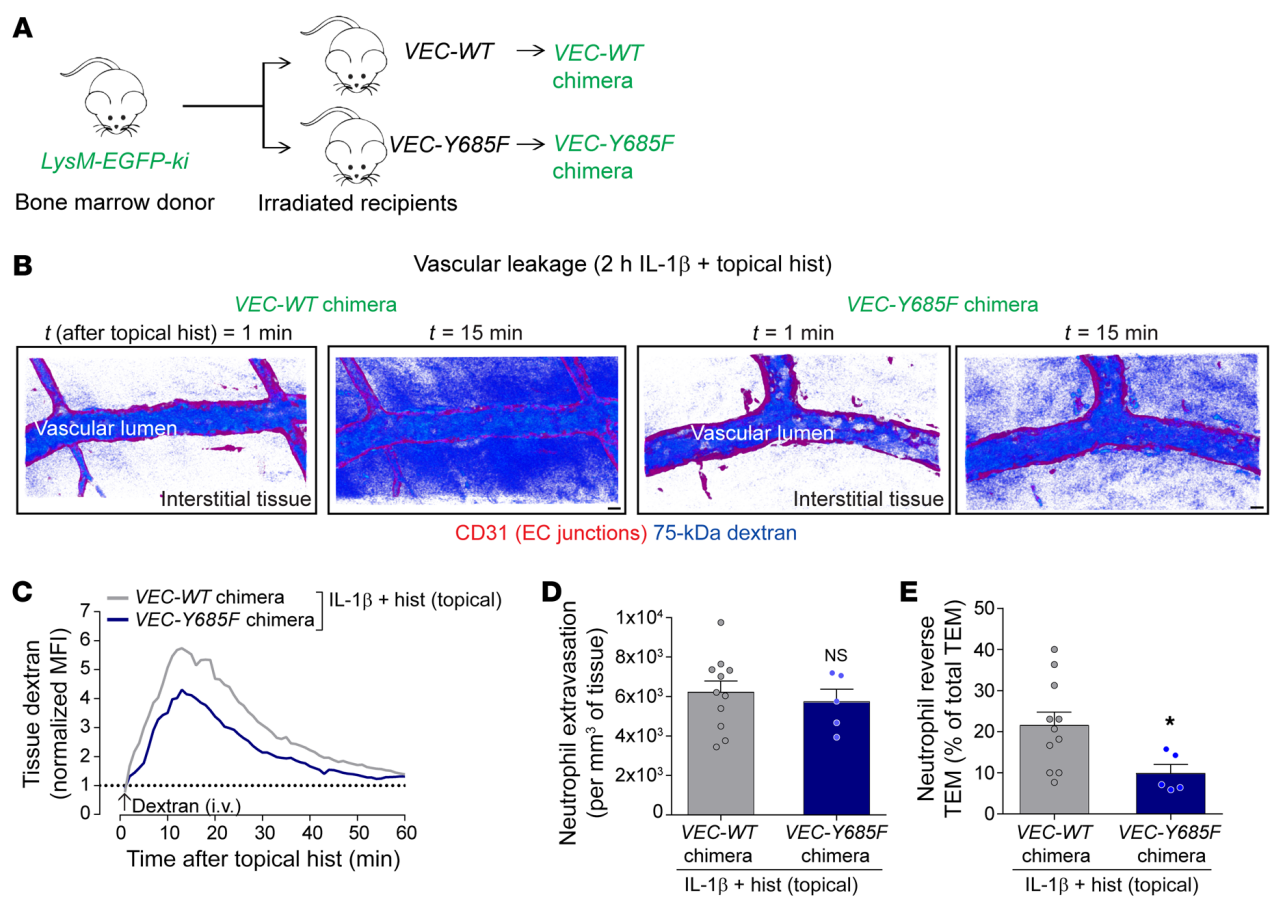
Chimeric VEC-Y658F mice exhibit reduced microvascular leakage induction and neutrophil reverse TEM. (**A**) Generation of chimeric mice exhibiting *LysM-EGFP-ki* hematopoietic cells within *VEC-WT* or *VEC-Y658F* recipients. (**B**) Representative confocal IVM images of postcapillary venular segments (stained with anti-CD31; red) subjected to IL-1β plus histamine stimulation at 2 time points after application of histamine in chimeric *VEC-WT* and *VEC-Y658F* mice, illustrating dextran leakage (blue pseudocolor intensity). Scale bars: 10 μm. (**C**) Time course of dextran accumulation in the perivascular region of selected IL-1β–stimulated postcapillary venules in *VEC-WT* and *VEC-Y658F* chimeric mice after topical application of histamine (*n* = 6–12 mice per group). (**D**) Total neutrophil extravasation (*n* = 5–11 mice per group). (**E**) Frequency of neutrophil reverse TEM events in relation to total TEM events of 16.6 ± 2.1 (*VEC-WT*) and 19.2 ± 3.1 (*VEC-Y685F*) per 300-μm venular segment within 2-hour microscopy periods (mean ± SEM, *n* = 5–11 mice). Data are represented as mean ± SEM (each symbol represents 1 mouse/independent experiment). Indicated statistical differences are shown by *P < 0.05, 2-tailed Student’s *t* test. NS, not significant.

To further investigate the causal link between increased microvascular leakage and disrupted neutrophil TEM, we took advantage of a knockin mouse model that expresses a Y685F mutant of VE-cadherin (*VEC-Y685F*) and shows reduced vascular leakage but normal neutrophil extravasation (9). Knockin mice expressing wild-type (WT) VE-cadherin (*VEC-WT*) were used as controls. To facilitate simultaneous quantification of neutrophil TEM dynamics and vascular leakage, chimeric mice were generated through adoptive transfer of bone marrow from *LysM-EGFP-ki* donor mice into irradiated *VEC-WT* and *VEC-Y685F* recipients (Figure 3A). Using the model of local IL-1β plus histamine, *VEC-Y685F* chimeric mice showed reduced (~26%) interstitial dextran accumulation (Figure 3, B and C) with no significant change in total neutrophil extravasation over 2 hours (Figure 3D), as compared with responses detected in chimeric *VEC-WT* controls. These results are in line with the findings of Wessel and colleagues using the Miles assay in which 36% suppression of histamine-induced vascular leakage was detected in the dorsal skin of *VEC-Y685F* mice (9). Of note, in agreement with our previous results (Figure 2D), IL-1β plus histamine caused a substantial frequency of neutrophil rTEM (~22%; Figure 3E) in *VEC-WT* chimeric mice. However, mice harboring the mutant form of VE-cadherin exhibited a significantly reduced frequency of this response (down to ~10%), culminating in approximately 55% inhibition of rTEM as compared with *VEC-WT* mice (Figure 3E). Together, our results provide compelling evidence for the ability of increased vascular leakage to disrupt luminal-to-abluminal motility of neutrophils through EC junctions resulting in neutrophil rTEM back into the vascular lumen.

### Microvascular leakage promotes trafficking of interstitial chemokines into the bloodstream.

We next explored the mechanism through which vascular leakage induction promotes disrupted directionality of neutrophil TEM. Because our earlier works identified reduced EC junctional expression of JAM-C as a driver of incomplete and reverse modes of neutrophil TEM (14, 15), this possibility was evaluated in the context of vascular leakage. However, local histamine, IL-1β, or the combination of both mediators had no impact on expression of EC JAM-C (Supplemental Figure 2). In considering alternative mechanisms, we hypothesized that elevated microvascular permeability may alter the local chemotactic gradient across venular walls. To explore this notion, because IL-1β is an effective inducer of a potent neutrophil chemokine CXCL1 (18, 19), we sought to investigate the impact of increased vascular leakage on generation and localization of CXCL1 in IL-1β–stimulated tissues.

**Figure 4:**
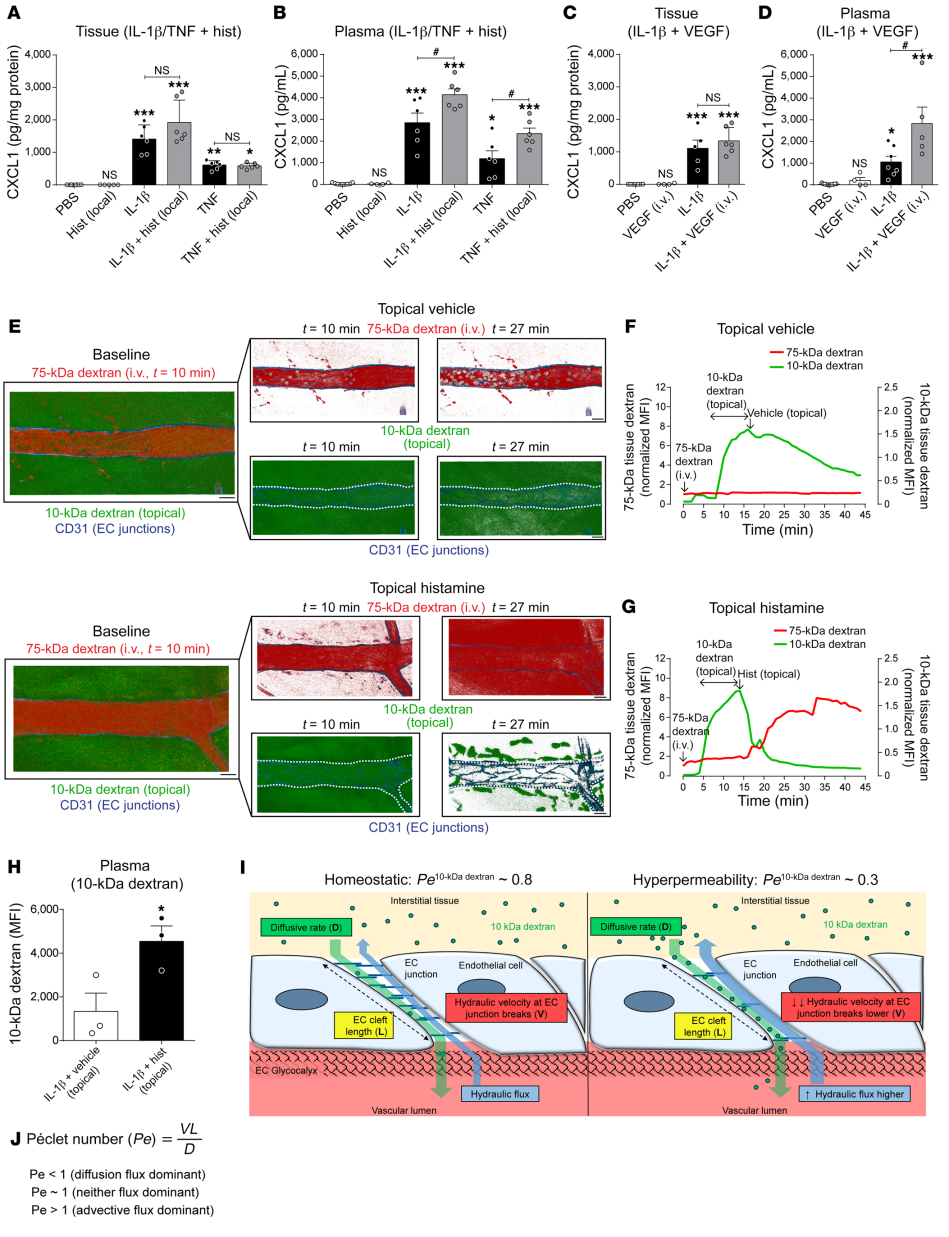
Stimulated microvascular leakage induces movement of small-molecular-weight proteins into the vascular lumen. (**A**–**D**) Cremaster muscles of WT mice were stimulated with IL-1β or TNF for 2 hours followed by local injection of histamine or i.v. VEGF (or corresponding vehicle), for 30 minutes before collection of tissue and plasma samples for quantification of CXCL1 levels by ELISA. Mice subjected to PBS, histamine, or VEGF alone were used as control (*n* = 4–7 mice/group). (**E**) Representative images of selected IL-1β–stimulated postcapillary venules showing movement of i.v. 75-kDa TRITC-dextran (marker of vascular leakage; red) and topically applied 10-kDa AF488-dextran (superfused for 10 minutes; green) after topical superfusion of PBS vehicle (top panels) or histamine (30 μM, lower panels; see Supplemental Video 5). Dashed lines indicate blood vessel borders. Scale bars: 20 μm. (**F** and **G**) Time course of 75-kDa TRITC-dextran and 10-kDa AF488-dextran accumulation in the perivascular region of selected IL-1β–stimulated postcapillary venules after superfusion of vehicle (**F**, *n* = 3 mice) or histamine (**G**, *n* = 3 mice). (**H**) Quantification of 10-kDa AF488-dextran in plasma samples of mice treated as detailed above (*n* = 3 mice/group). (**I** and **J**) Schematic diagram and mathematical equation of the Péclet number (*Pe*) (see Supplemental Figure 4). Data are represented as mean ± SEM (each symbol represents 1 mouse/independent experiment). Statistically significant differences from PBS (**A**–**D**) or IL1β plus vehicle (**H**) are indicated by *P < 0.05; **P < 0.01; ***P < 0.001, between groups as indicated by lines or by ^#^*P* < 0.05, 1-way ANOVA followed by Bonferroni’s post hoc test or 2-tailed Student’s *t* test. NS, not significant.

IL-1β stimulation of WT cremaster muscles led to a robust increase in tissue and plasma CXCL1, as compared with PBS-treated mice (Figure 4, A–D). Although locally injected histamine or i.v. VEGF had no significant effect on IL-1β–induced tissue levels of CXCL1, they elevated the associated plasma levels of CXCL1 (Figure 4, A–D). Of note, the vasoactive agents on their own did not induce either tissue or plasma CXCL1 (Figure 4, A–D). Similarly, as found with IL-1β, TNF stimulation of cremaster muscles increased tissue and plasma CXCL1, with the latter being further elevated after local administration of histamine (Figure 4, A and B). In addition, increased tissue and plasma CXCL1 were detected in mice subjected to the hyperpermeability-inducing cremasteric IR injury (Supplemental Figure 3). These results imply that vascular permeability–enhancing stimuli can promote the trafficking of endogenously generated CXCL1 from the tissue into the bloodstream. To directly investigate this possibility, we asked a general but fundamental question: can an extravascular protein, comparable to the size of a chemokine (~8 kDa), traffic into the bloodstream through hyperpermeable leaky microvessels? To address this, we examined by confocal IVM the localization of topically applied small-MW (10 kDa) AF488-dextran in control and histamine-treated IL-1β–stimulated cremaster muscles. Here, vascular leakage was simultaneously quantified using i.v.-injected high-MW (75 kDa) TRITC-dextran. Superfusion of exteriorized cremaster muscles with 10-kDa AF488-dextran (for 10 minutes) led to its rapid and sustained accumulation in the extravascular space surrounding blood vessels (Figure 4, E and F, and Supplemental Video 5). Topical application of histamine caused immediate leakage of blood-circulating 75-kDa TRITC-dextran into the interstitium (Figure 4, E and G, and Supplemental Video 5), confirming induction of vascular leakage. Importantly, this effect was associated with rapid (<5 minutes) disappearance of the 10-kDa AF488-dextran signal from the perivascular space, as compared with tissues treated with topical vehicle (Figure 4, E–G). Furthermore, in mice treated with topical histamine, a significant level of 10-kDa AF488-dextran was detected in plasma (Figure 4H).

Collectively, these results demonstrate that under conditions of enhanced vascular permeability, a small-MW protein can diffuse from the interstitium into the bloodstream against an advective flow in the opposite direction. As seemingly unexpected, further validation of these experimental findings were sought through mathematical modeling. Briefly, as the molecular flux across the vessel wall is determined by a combination of diffusive and advective transport, for a small-MW protein in the extravascular compartment (e.g., 10-kDa dextran or a chemokine) to enter the blood circulation, the diffusive flux from tissue to blood must be high enough to overcome the opposing advective flux (Figure 4I). This ratio is defined by the Péclet number (*Pe*) (Figure 4J). If the *Pe* is substantively less than 1, then there can be diffusion against an advective flow of fluid. If the *Pe* is more than 1, then diffusion cannot effectively oppose filtration — in other words, for a molecule to diffuse against a flow of fluid *Pe* needs to be less than 1. Using previously published values for the hydraulic conductivity properties of ECs, and known diffusion coefficients, the *Pe* number for a 10-kDa dextran in cremaster muscle microvessels is calculated to be approximately 0.8 under normal conditions (Supplemental Figure 4). Such a scenario would allow some diffusion of interstitial protein into the vascular lumen, in line with our experimental data (Figure 4H). However, under conditions of increased vascular permeability (e.g., as induced by local histamine) this falls substantially to values at which diffusion dominates advective flux (i.e., *Pe* < 0.3; Figure 4I and Supplemental Figure 4). This modeling of the molecular flux across cremasteric venular walls supports our experimental data and provides additional endorsement for the concept that vascular hyperpermeability can facilitate the trafficking of extravascular small molecules into the vascular lumen.

**Figure 5:**
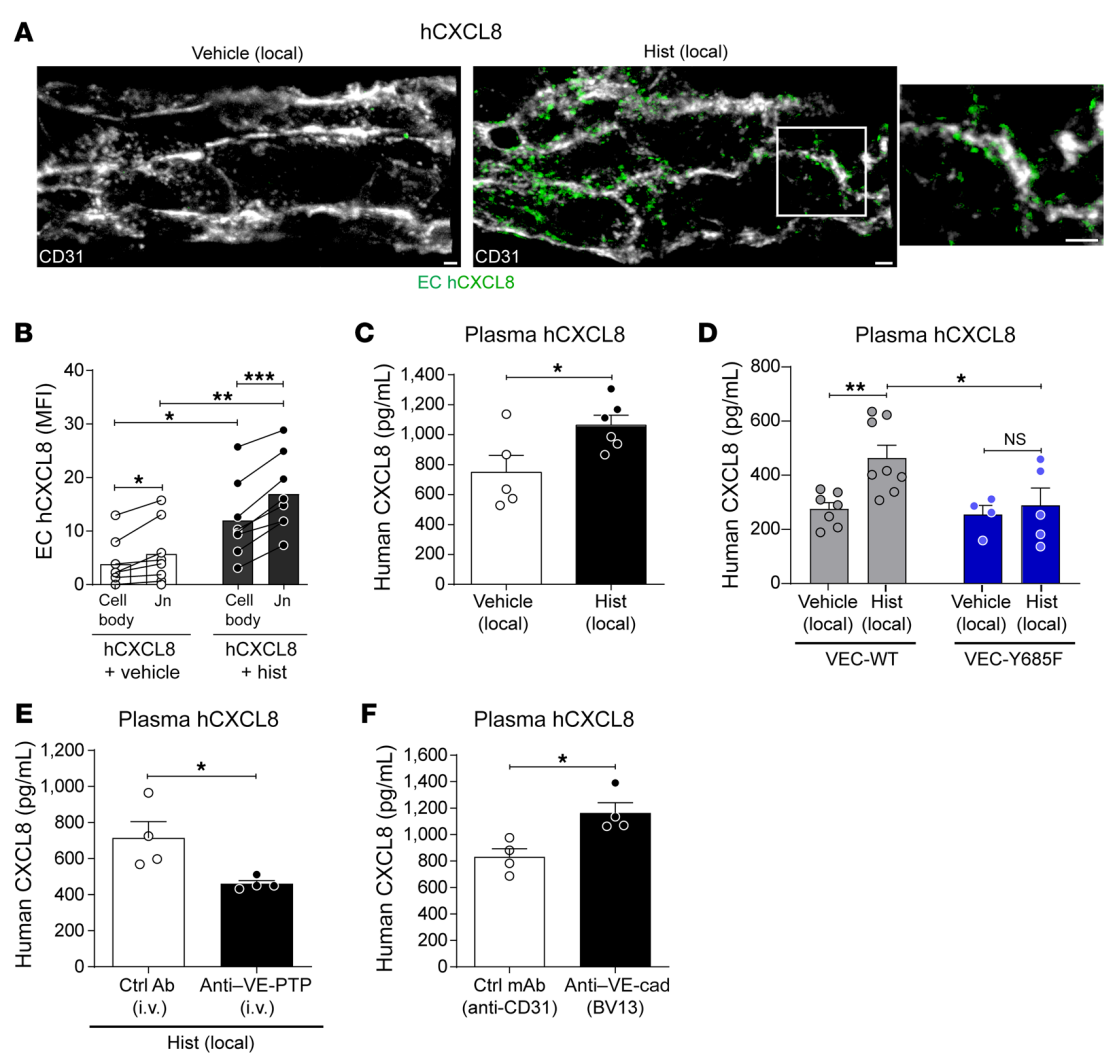
Vascular leakage induction promotes trafficking of tissue chemokine through venular walls. Cremaster muscles were injected locally with IL-1β (50 ng) and human CXCL8 (hCXCL8, 500 ng) for 1 hour, followed by i.s. injection of vehicle (control) or histamine for an additional 1 hour. (**A**) Representative confocal images of postcapillary venules stained for hCXCL8 within an EC (labeled by anti-CD31) isosurface mask with an enlarged image of the boxed region. Scale bars: 2 μm. (**B**) Quantification of hCXCL8 MFI on EC body and junctions (*n* = 8 mice/group). (**C**–**F**) Plasma levels of locally applied hCXCL8 in WT mice (**C**), *VEC-WT* and *VEC-Y685F* mice (**D**), in mice treated with a control or anti–VE-PTP Ab (200 μg, i.v.) (**E**), or treated with an anti–VE-cadherin mAb (i.s. BV13, 100 μg), or a control mAb (nonblocking anti–CD31 mAb, 100 μg) for 3 hours before local administration with hCXCL8 for 1 hour (*n* = 4–8 mice/group) (**F**). Data are represented as mean ± SEM (each symbol represents 1 mouse/independent experiment). Statistically significant differences between cell body vehicle versus cell body histamine and EC junction (Jn) vehicle versus Jn histamine groups were analyzed by 1-way ANOVA followed by Bonferroni’s post hoc test (*P < 0.05; **P < 0.01). Differences between the other indicated groups are shown by *P < 0.05; **P < 0.01; ***P < 0.001 analyzed by 2-way ANOVA followed by Bonferroni’s post hoc test (**B** and **D**) or 2-tailed paired (**B**) or unpaired Student’s *t* test (**C**, **E**, and **F**). NS, not significant.

We next directly assessed the capacity of an interstitial chemokine to engage with EC junctions and to leak from the tissue into the bloodstream after vascular permeability induction. For this purpose, we analyzed the localization of human CXCL8 (hCXCL8) in relation to ECs, when locally applied to IL-1β–stimulated cremaster muscles, in the presence or absence of histamine. Although in control tissues the chemokine was modestly aligned with the endothelium, this was significantly elevated after local application of histamine, most notably in relation to EC junctions (Figure 5, A and B). Furthermore, while locally injected hCXCL8 could be detected in plasma of mice treated with IL-1β only, this response was significantly increased (~42%) in mice treated with IL-1β plus histamine (Figure 5C). A similar increase in plasma levels of locally injected hCXCL8 was noted in mice treated with IL-1β plus VEGF as compared with animals treated with IL-1β plus vehicle (Supplemental Figure 5). Furthermore, VEC-Y685F mutant mice that exhibit a defect in vascular permeability induction (ref. 9 and Figure 3, B and C) showed reduced plasma levels of hCXCL8 as compared with levels detected in control VEC-WT animals (Figure 5D). The link between vascular permeability induction and tissue-to-blood chemokine movement was further investigated through the use of blocking anti–VE-PTP and anti–VE-cadherin Abs. Specifically, an anti–VE-PTP Ab that inhibits vascular permeability induction through activation of Tie2 (20, 21) (a) significantly suppressed histamine-induced vascular leakage in the mouse cremaster muscle (Supplemental Figure 6A), and (b) significantly reduced plasma levels of locally administered hCXCL8 in mice treated with IL-1β plus histamine, as compared with control Ab–treated mice (Figure 5E). Animals treated with the anti–VE-PTP Ab also showed reduced plasma levels of endogenously generated CXCL1, as compared with levels detected in control Ab–treated mice (Supplemental Figure 6B). In contrast to the inhibitory effects of the anti–VE-PTP Ab, a blocking anti–VE-cadherin mAb (clone BV13) (22) that promoted vascular leakage induction in the mouse cremaster muscle (Supplemental Figure 6, C and D) enhanced plasma levels of locally administered hCXCL8 as compared with levels detected in mice treated with a control mAb (anti-CD31) (Figure 5F).

Together, through experimental and mathematical modeling, we provide compelling evidence to show that the loosening of EC junctions during increased vascular permeability can promote the mobilization of small-MW proteins from the interstitial tissue to the bloodstream and thus influence the compartmentalization of extravascular chemokines.

### Luminal CXCL1 promotes neutrophil rTEM.

**Figure 6:**
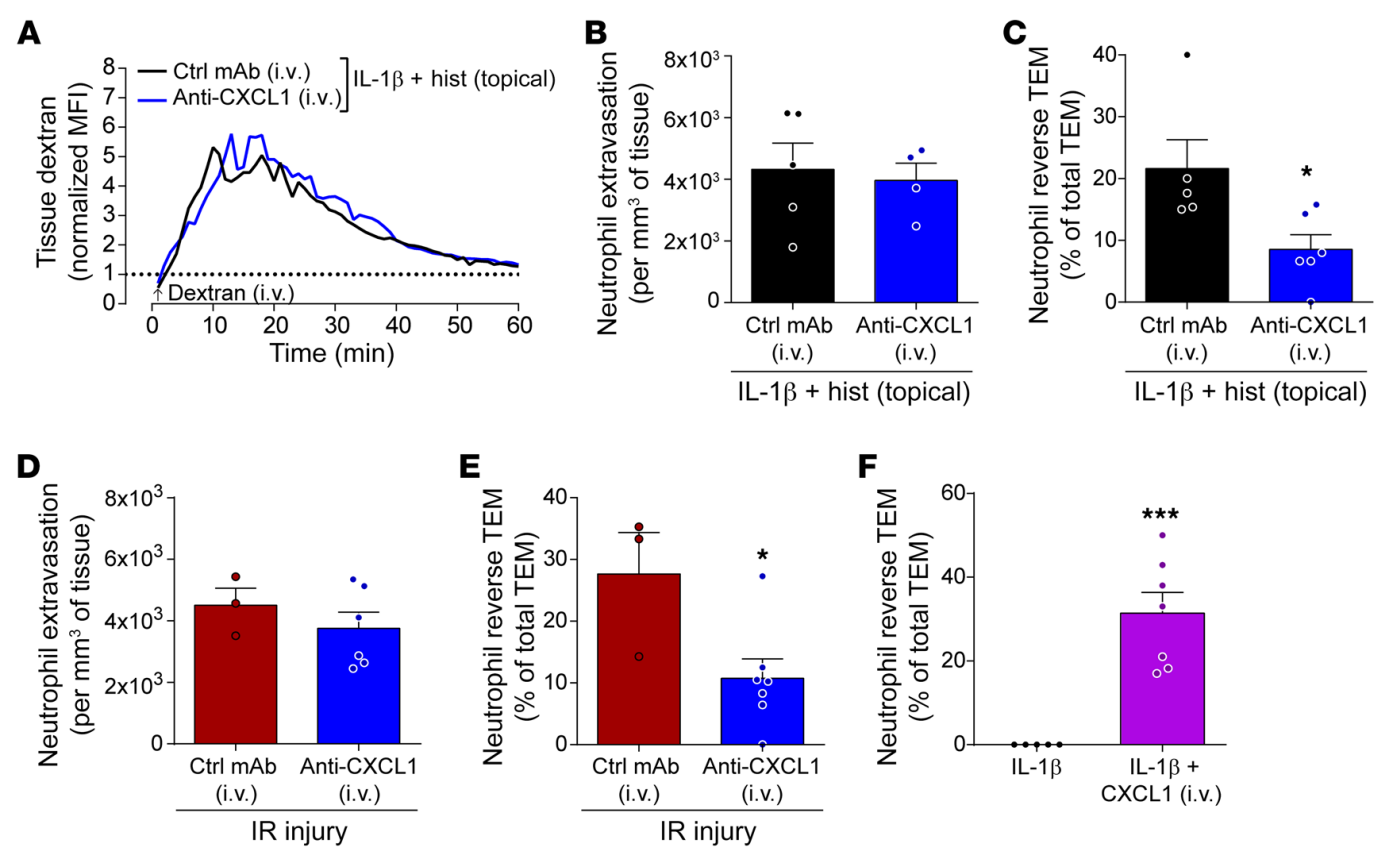
Systemic CXCL1 promotes neutrophil reverse TEM. Cremaster muscles of *LysM-EGFP-ki* mice were stimulated with IL-1β (50 ng for 2 hours) followed by topical superfusion of histamine onto exteriorized tissues or subjected to IR injury. Blocking anti-CXCL1 mAb (1 mg/kg), or control IgG, was injected i.v. 30 minutes before exteriorization of tissues or at the point of tissue reperfusion. (**A**) Time course of dextran accumulation in the perivascular region of selected stimulated postcapillary venules. Tissue dextran accumulation is represented as normalized MFI (*n* = 4–6 mice per group). (**B**) Total neutrophil extravasation (*n* = 4–5 mice/group) and (**C**) frequency of neutrophil reverse TEM events in relation to total TEM events of 14 ± 2 (control mAb) and 20 ± 2.9 (anti-CXCL1 mAb) per 300-μm venular segment within 2-hour microscopy periods (mean ± SEM, *n* = 5–6 mice/group). (**D**) Total neutrophil extravasation (*n* = 3–6 mice/group) and (**E**) frequency of neutrophil reverse TEM events in relation to total TEM events of 26.7 ± 3.8 (control mAb) and 26 ± 4.3 (anti-CXCL1) per 300-μm venular segment within 2-hour microscopy periods (mean ± SEM, *n* = 3–7 mice/group). (**F**) Cremaster muscles of *LysM-EGFP-ki* mice were stimulated with IL-1β (50 ng for 2 hours) followed by i.v. injection of murine rCXCL1 (50 ng). Frequency of neutrophil reverse TEM events in relation to total TEM events of 16 ± 2.8 (IL-1β) and 12.9 ± 1.8 (IL-1β + CXCL1) per 300-μm venular segment within 2-hour microscopy periods (mean ± SEM, *n* = 5–7 mice/group). Data are represented as mean ± SEM (each symbol represents 1 mouse/independent experiment). Statistically significant differences from control mAb–treated groups (**C** and **E**) or IL-1β–treated tissues (**F**) are shown by *P < 0.05; ***P < 0.001, 2-tailed Student’s *t* test.

We next investigated the functional impact of increased plasma CXCL1 on neutrophil TEM. For this purpose, 2 hours after stimulation of cremaster muscles with IL-1β, *LysM-EGFP-ki* mice were injected i.v. with a blocking anti-CXCL1 mAb before topical application of histamine. Although at the dose employed i.v. administration of the anti-CXCL1 mAb had no impact on plasma protein leakage or total neutrophil extravasation (Figure 6, A and B), this intervention significantly reduced the frequency of neutrophil rTEM events (~60% inhibition), as compared with control Ab–injected mice (Figure 6C). Similarly, systemic CXCL1 blockade had no impact on total neutrophil extravasation in cremaster muscles subjected to IR insult (Figure 6D) but suppressed neutrophil rTEM in this permeability-enhancing reaction (~60% inhibition; Figure 6E). Because this suggested that luminal CXCL1 can drive neutrophil rTEM, this was categorically investigated through i.v. injection of exogenous CXCL1 in *LysM-EGFP-ki* mice stimulated locally with IL-1β (2 hours). Within this protocol, i.v. CXCL1 promoted a substantial frequency of neutrophil rTEM through cremasteric venules (~30%; Figure 6F), whereas as noted previously (Figure 1E), local IL-1β on its own did not cause neutrophil rTEM (Figure 6F). Collectively, the present results unequivocally demonstrate that vascular CXCL1 can drive neutrophil TEM toward the luminal aspect of the endothelium.

### Development of a cell labeling strategy for tracking of neutrophil rTEM in vivo.

**Figure 7:**
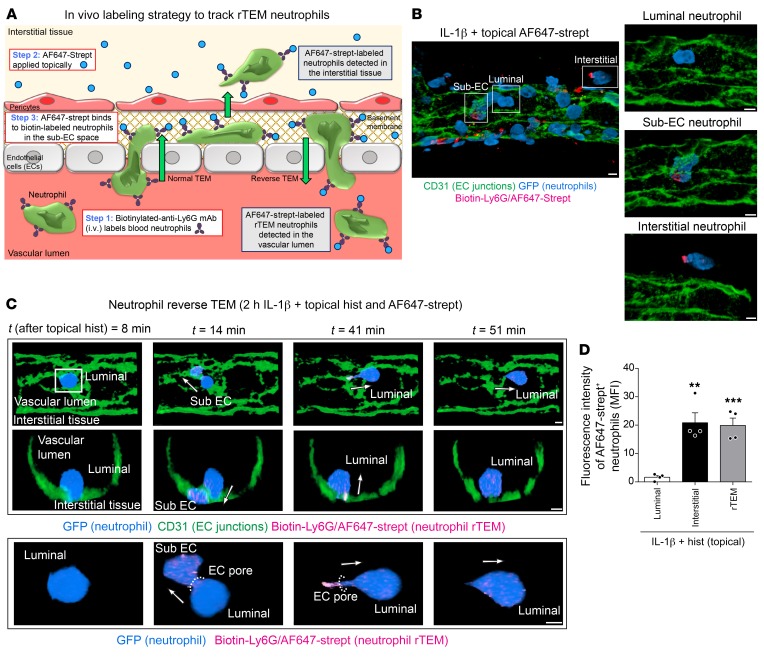
Development of an in vivo labeling strategy for tracking rTEM neutrophils. (**A**) Diagram detailing the labeling method. Cremaster muscles of *LysM-EGFP-ki* mice were stimulated with IL-1β (50 ng for 2 hours) followed by an i.v. injection of biotinylated anti-Ly6G (2 μg) to label blood neutrophils at *t* = 90 minutes. The tissues were superfused with histamine (30 μM) or vehicle in conjunction with AF647-streptavidin (1 μg/mL) for 2 hours. (**B**) Representative confocal image of an IL-1β–stimulated postcapillary venule illustrating the extent of AF647-streptavidin labeling of neutrophils at different stages of trafficking (left panel). Right panels shown enlarged images of the boxed regions and illustrate examples of AF647-streptavidin^–^ luminal, AF647-streptavidin^+^ sub-EC, and AF647-streptavidin^+^ interstitial neutrophils. Scale bars: 5 μm. (**C**) Representative confocal IVM images of a tissue stimulated with IL-1β plus histamine (see Supplemental Video 6) illustrating the effective labeling of an rTEM event. The exemplified neutrophil shows that once the cell has breached an EC junction, the leading body part in the sub-EC space rapidly becomes AF647-streptavidin^+^ while the luminal body segment remains AF647-streptavidin^–^. The AF647-streptavidin^+^ neutrophil can be easily tracked as it migrates in a reverse manner toward the vascular lumen and reenters the bloodstream. Luminal and cross-sectional views are shown with the arrows indicating the direction of motility of the indicated neutrophil. Scale bars: 3 μm. (**D**) Fluorescence intensity of AF647-streptavidin on neutrophils in the venular lumen, tissue, and cells exhibiting rTEM (*n* = 4 mice/group) during an IL-1β plus histamine reaction. Data are represented as mean ± SEM (each symbol represents 1 mouse/independent experiment). Statistically significant differences from luminal neutrophils are shown by **P < 0.01; ***P < 0.001, 1-way ANOVA followed by Bonferroni’s post hoc test.

Aiming to gain insight into the fate, phenotype, and pathophysiological relevance of rTEM neutrophils stemming from a local hyperpermeable inflammatory site, we established a method for tracking of these cells. Previous studies employing zebrafish and murine models of tissue injury have utilized genetically encoded photolabeling protocols to track reverse-migrating neutrophils from within injured interstitial tissues back into the vascular lumen and into distal organs (23–25). However, as the neutrophil reverse migration phenomenon noted in the present study is restricted to breaching of the endothelium only, photoconverting and photoactivation of neutrophils confined within tight EC junctions or the thin sub-EC space (<3 μm wide) renders such genetic strategies inappropriate for exclusive delineation of rTEM cells (e.g., preliminary works with transgenic mice expressing the Kaede photoconvertible fluorescence protein tracked fewer than 10 rTEM neutrophils/inflamed tissue; data not shown). Hence, as part of the present study we have developed a labeling method that crisply delineates neutrophils that reverse migrate within EC junctions and reenter the vascular lumen (Figure 7A). The method takes advantage of the strong affinity of streptavidin for biotin and our observation that locally applied streptavidin is retained within the cremaster muscle tissue and does not move into the systemic circulation, even under conditions of increased vascular permeability (Supplemental Figure 7A). Initial experiments applied this labeling method to staining of neutrophils in IL-1β–stimulated tissues. Briefly, after local application of IL-1β (1.5 hours), *LysM-EGFP-ki* mice were injected i.v. with biotinylated anti-Ly6G mAb, a step that as anticipated selectively labeled over 99% of all circulating neutrophils (Supplemental Figure 7B). Thirty minutes later, cremaster muscles were surgically exteriorized and tissues were topically superfused with AF647-streptavidin. Analysis of tissues by confocal microscopy showed that with this protocol luminal neutrophils were GFP^+^ but streptavidin^–^, whereas neutrophils in the sub-EC space and in the interstitial tissue were clearly positive for both GFP and streptavidin (Figure 7B). We next sought to investigate the ability of this labeling method to track rTEM neutrophils during the hyperpermeability reaction to IL-1β plus histamine where histamine and AF647-streptavidin were simultaneously superfused onto surgically exteriorized tissues. Here, intriguingly we noted that in neutrophils exhibiting TEM, leading protrusions in the sub-EC space rapidly became intensely streptavidin^+^ (Figure 7C), resulting in effective labeling of all neutrophils that partially or fully breached the endothelium. This included cells that completely breached the venular wall and entered the surrounding interstitial tissue as well as cells that reverse migrated from within EC junctions or the sub-EC space back into the vascular lumen (Figure 7, A–D). Similar results were obtained when AF647-streptavidin was injected locally into cremaster muscles (400 ng for 2 hours) as opposed to being topically superfused (Supplemental Figure 7, C and D). Importantly, unlabeled mice and mice subjected to the biotin-streptavidin labeling strategy exhibited similar levels of neutrophil migration into tissues and neutrophil rTEM (Supplemental Figure 8, A and B, as compared with Figure 2, A and D) as well as similar neutrophil migration velocity within EC junctions and the interstitial tissue (Supplemental Figure 8, C and D).

Thus, we have established a cell labeling technique for direct tracking of neutrophils that exhibit rTEM in vivo. This methodological advancement enables definitive explorations into the phenotype and fate of rTEM neutrophils.

### rTEM neutrophils disseminate into the systemic and pulmonary circulation and exhibit an activated phenotype.

**Figure 8:**
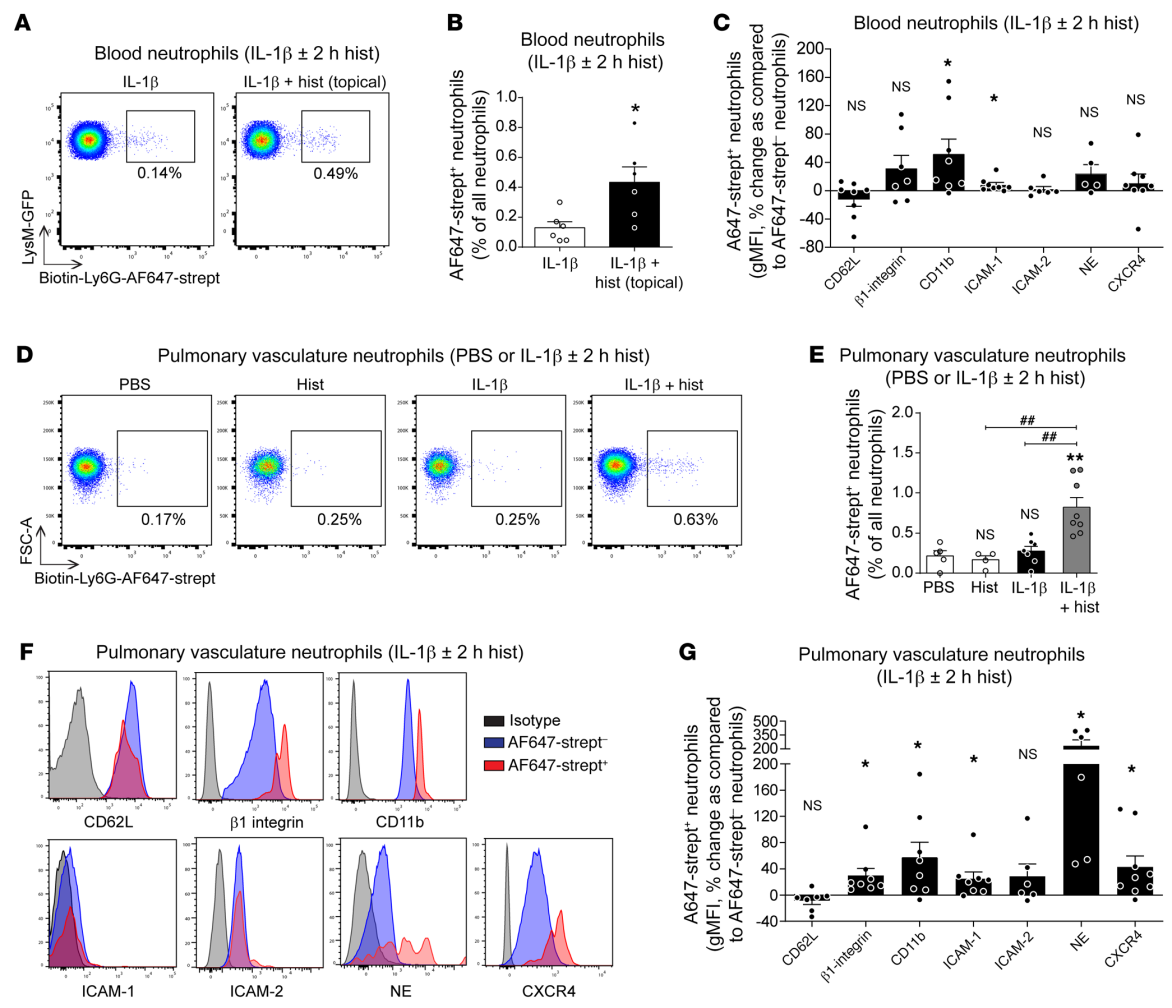
Labeled rTEM neutrophils are present in blood and pulmonary vasculature and show an activated phenotype. (**A** and **B**) *LysM-EGFP-ki* mice were subjected to local cremaster muscle stimulation with IL-1β (2 hours) and an i.v. injection of biotinylated anti-Ly6G (2 μg) at *t* = 90 minutes. The tissues were then superfused with histamine (30 μM) or vehicle in conjunction with AF647-streptavidin (1 μg/mL) for 2 hours. Peripheral blood was analyzed for the frequency of AF647-streptavidin^+^ neutrophils. (**A**) Representative flow cytometry profiles and (**B**) frequency of AF647-streptavidin^+^ neutrophils (LysM-GFP^hi^Gr-1^hi^) (*n* = 6 mice/group). (**C**–**G**) WT mice were subjected to cremaster muscle stimulation with IL-1β or PBS for 2 hours followed by i.v. injection of biotinylated anti-Ly6G (2 μg) at *t* = 90 minutes. The mice then received an i.s. injection of AF647-streptavidin (400 ng) coadministered with histamine (200 μL of 30 μM solution) or PBS for 2 hours. Peripheral blood and pulmonary vascular washout samples were analyzed by FACS. (**C**) Expression of indicated markers on AF647-streptavidin^+^ neutrophils relative to levels on AF647-streptavidin^–^ neutrophils in blood samples collected from mice subjected to IL-1β plus histamine, as measured by geometric MFI (gMFI) (*n* = 5–8 mice/group). (**D**) Representative flow cytometry profiles and (**E**) frequency of pulmonary vascular washout AF647-streptavidin^+^ neutrophils (Gr-1^hi^CD115^–^) (*n* = 4–8 mice/group). (**F**) Representative FACS histograms and (**G**) expression of indicated markers on AF647-streptavidin^+^ neutrophils relative to levels on AF647-streptavidin^–^ neutrophils in pulmonary vascular washout samples collected from mice stimulated with IL-1β plus histamine, as measured by gMFI (*n* = 6–9 mice/group). Data are represented as mean ± SEM (each symbol represents 1 mouse/independent experiment). Statistically significant differences from IL-1β (**B**), gMFI of indicated markers of blood AF647-streptavidin^–^ neutrophils (**C**), PBS (**E**), and gMFI of indicated markers on pulmonary vascular washout AF647-streptavidin^–^ neutrophils (**G**) are shown by *P < 0.05; **P < 0.01 or by indicated comparisons ^##^*P* < 0.01, 2-tailed Student’s *t* test (**B**, **C**, and **G**) or 1-way ANOVA followed by Bonferroni’s post hoc test (**E**). NS, not significant.

Exploiting our labeling method, we next investigated the distribution of rTEM neutrophils stemming from local hyperpermeability sites. Because our confocal IVM studies showed rTEM neutrophils to reenter the vascular lumen and rapidly detach from the luminal aspect of the endothelium (Supplemental Video 6), we initially sought to detect these cells in the systemic circulation. For this purpose, using mice subjected to cremasteric stimulation with IL-1β, blood samples were taken 2 hours after local administration of histamine or vehicle in conjunction with the new biotin-streptavidin labeling protocol. Flow cytometric analysis of samples from IL-1β–treated mice showed very low levels of streptavidin^+^ circulating blood neutrophils (~0.1%, corresponding to a total of approximately 800 streptavidin^+^ neutrophils/mL of blood); this number was significantly increased in blood samples of mice locally stimulated with IL-1β plus histamine (~0.4%, corresponding to approximately 2,800 streptavidin^+^ neutrophils/mL of blood) (Figure 8, A and B). Aligned with the substantive frequency of neutrophil rTEM seen in the corresponding reactions (see Figure 2D), these results strongly indicated the presence of rTEM neutrophils in the systemic circulation. Focusing on the hyperpermeability reaction to IL-1β plus histamine, we next explored the phenotype of streptavidin^+^ neutrophils as compared to streptavidin^–^ cells. Streptavidin^+^ blood neutrophils exhibited no significant change in expression of L-selectin (CD62L), β1 integrins, ICAM-2, neutrophil elastase (NE), or CXCR4 but showed significantly increased expression of CD11b, and a low but significant increase in expression of ICAM-1 (Supplemental Figure 9A and Figure 8C). Based on such an activated phenotype, and as guided by our previous works (14, 15), we hypothesized that streptavidin^+^ rTEM neutrophils may be additionally retained within the pulmonary vasculature. To address this possibility, we analyzed the percentage and phenotype of streptavidin^+^ neutrophils in pulmonary vasculature washouts of mice subjected to local cremaster muscle stimulations with PBS, histamine, IL-1β, or IL-1β plus histamine (2 hours) and biotin-streptavidin labeling. Although animals treated locally with PBS, histamine, or IL-1β showed similar levels of streptavidin^+^ neutrophils (~0.2%), mice stimulated with IL-1β followed by histamine for 2 hours showed an enrichment of streptavidin^+^ neutrophils in the pulmonary vasculature (~0.8%) (Figure 8, D and E). Increasing the local stimulation period with histamine to 4 hours resulted in a similar level of streptavidin^+^ neutrophils in blood (~0.42%) but led to a reduced and nonsignificant level in the pulmonary vasculature (~0.2%) (Supplemental Figure 9, B and C). Interestingly, in mice treated locally with IL-1β followed by 4 hours of stimulation with histamine, but not 2 hours, a significant level of streptavidin^+^ neutrophils was detected in the bone marrow (Supplemental Figure 9, D–G). Together, these results suggest that the retention of rTEM neutrophils in the lungs is transient and that this subset of cells eventually migrate to the bone marrow. Furthermore, as compared with streptavidin^–^ cells, streptavidin^+^ pulmonary vascular neutrophils showed no significant change in expression of L-selectin and ICAM-2 but exhibited a significant increase in expression of β1 integrins, CD11b, ICAM-1, NE, and CXCR4 (Figure 8, F and G). The latter is in line with the observed trafficking of the streptavidin^+^ cells to the bone marrow. Of note, in IL-1β plus histamine–treated mice subjected to our labeling protocol, streptavidin^–^ neutrophils in both blood and pulmonary vascular washout samples showed a similar phenotype to neutrophils acquired from unlabeled mice treated locally with just PBS, histamine, or IL-1β alone (Supplemental Figure 10, A and B). This crucial set of data precludes the possibility that in IL-1β plus histamine–treated mice circulating soluble factors determine the phenotype of the streptavidin^+^ cells. Additionally, these results suggest that the streptavidin^+^ rTEM neutrophils have no impact on the phenotype of streptavidin^–^ neutrophils.

In summary, we provide evidence for the ability of rTEM neutrophils stemming from a local hyperpermeability site to reenter the systemic circulation and to traffic to lungs, where they exhibit an activated proadhesive phenotype, before returning to the bone marrow.

### Disseminated rTEM neutrophils localize to sites of vascular leakage in lungs.

**Figure 9:**
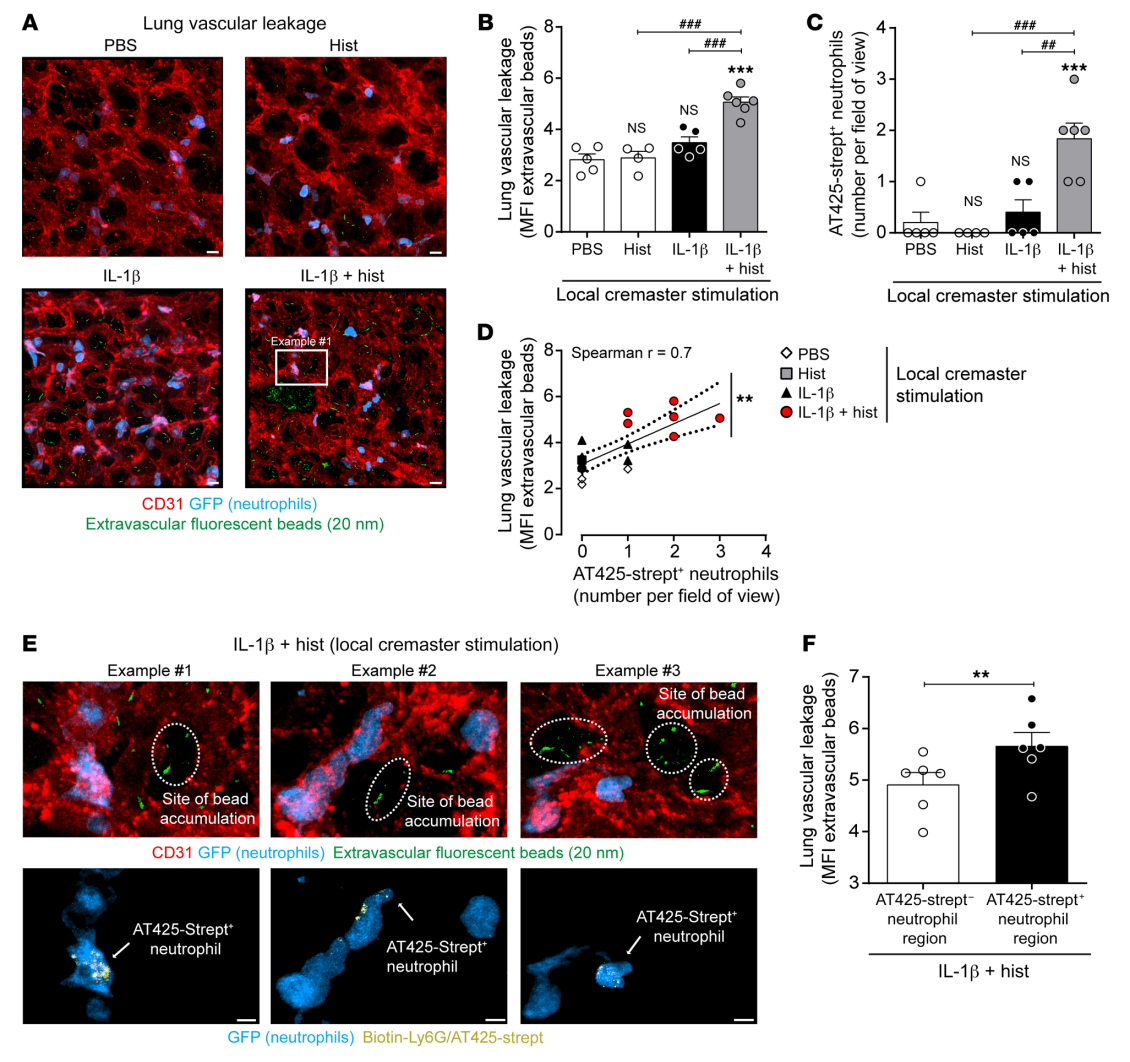
Accumulation of labeled rTEM neutrophils in lungs is linked with lung injury. The cremaster muscles of *LysM-EGFP-ki* mice were locally stimulated with IL-1β or PBS (2 hours). Mice were injected i.v. with AF555–anti-CD31 mAb (10 μg), biotin–anti-Ly6G mAb, and fluorescent microspheres (20 nm diameter, 9.1 × 10^13^ beads) at *t* = 90 minutes to label the vasculature and neutrophils, and to quantify vascular leakage, respectively. To induce and track rTEM neutrophils stemming from the cremaster muscles, tissues were additionally stimulated with locally applied histamine or vehicle (PBS) in combination with Atto425-streptavidin (AT425-Strept, 400 ng) for 120 minutes. The lungs were then analyzed by confocal microscopy. (**A**) Representative confocal images of alveolar capillaries in whole-mount imaged lungs of mice in which the cremaster muscles were locally stimulated as indicated (CD31-labeled vessels, red; neutrophils, blue; extravascular beads, green). Scale bars: 20 μm. (**B**) Lung vascular leakage as quantified by accumulation (MFI) of extravascular beads (*n* = 4–6 mice/group) and (**C**) number of AT425-streptavidin^+^ neutrophils per field of view (*n* = 4–6 mice per group), in mice subjected to indicated cremaster muscle stimulations. (**D**) Correlation of the number of AT425-streptavidin^+^ neutrophils and extravascular beads in lung tissues (*n* = 4–6 mice/group). Line indicates linear regression and dashed lines 95% confidence band (Spearman’s *r* = 0.7). (**E**) High-magnification images of lung sections showing AT425-streptavidin^+^ neutrophils in close apposition to sites of extravascular fluorescent bead accumulation. Scale bars: 3 μm. (**F**) Lung vascular leakage in close proximity (<60 μm) of AT425-streptavidin^–^ neutrophils or AT425-streptavidin^+^ neutrophils (*n* = 6 mice/group). Data are represented as mean ± SEM (each symbol represents 1 mouse/independent experiment). Statistically significant differences from PBS or indicated group are shown by **P < 0.01; ***P < 0.001, and between indicated groups by ^##^*P* < 0.01; ^###^*P* < 0.001, 1-way ANOVA followed by Bonferroni’s post hoc test (**B** and **C**), or Spearman’s rank correlation test (**D**), or paired *t* test (**F**). NS, not significant.

**Figure 10:**
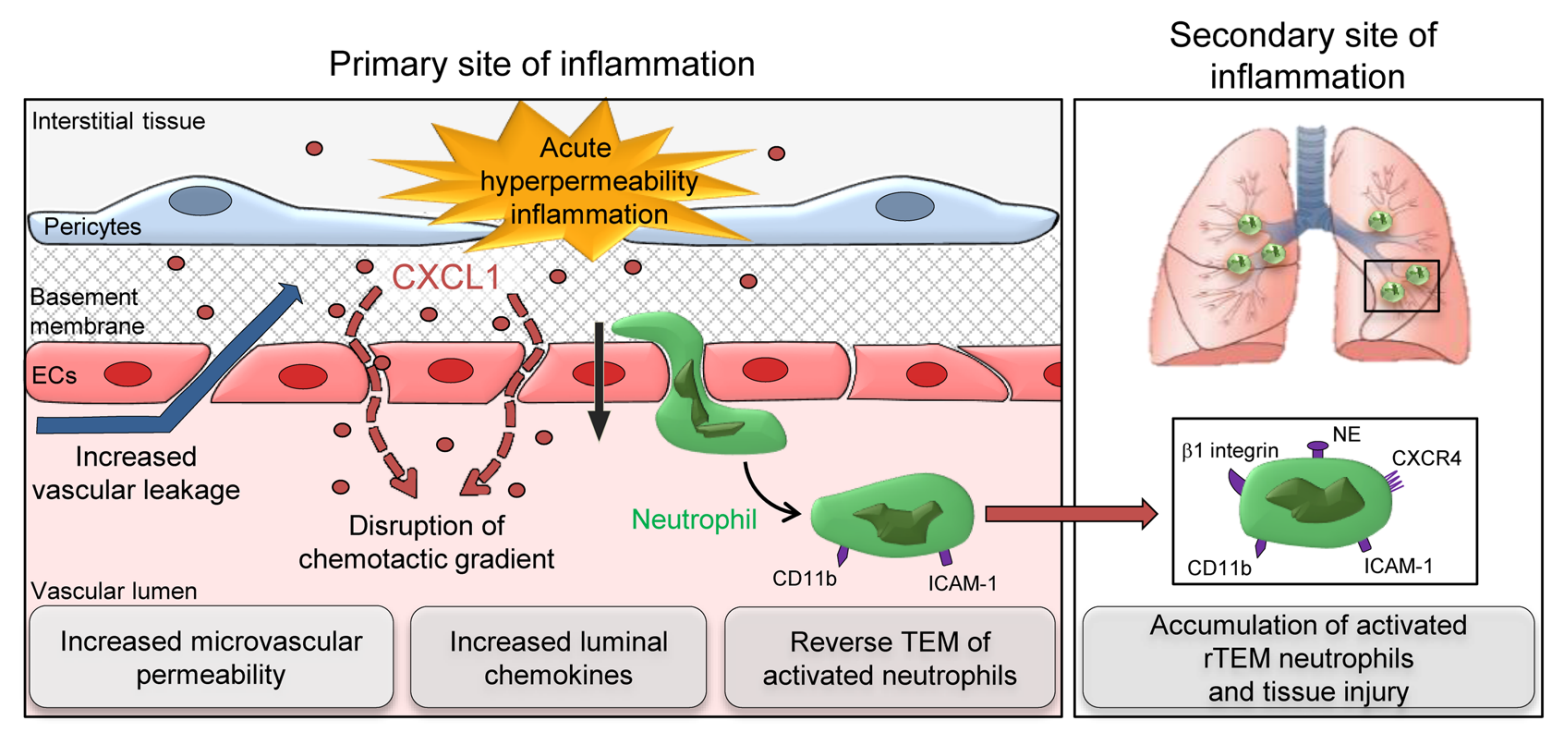
Schematic diagram of the cascade of events that link a local hyperpermeability inflammatory reaction to neutrophil rTEM and development of lung injury.

We have previously shown an association between rTEM neutrophils stemming from local inflammatory sites characterized by reduced junctional expression of EC JAM-C and remote organ injury (14, 15). We therefore hypothesized that neutrophil rTEM driven by local hyperpermeability could similarly cause distant organ damage. To investigate this notion, pulmonary vascular leakage resulting from stimulation of cremaster muscles was assessed by measuring sub-EC and extravascular accumulation of intravenously administered fluorescent microspheres (26). Furthermore, to investigate if this response was associated with accumulation of rTEM neutrophils stemming from the cremaster muscle, the experiments incorporated the new biotin-streptavidin labeling protocol. Our findings showed that mice subjected to cremaster muscle stimulation with IL-1β plus histamine, but neither stimulus on its own or local PBS, exhibited lung vascular leakage (Figure 9, A and B). In addition, although all reactions tested exhibited similar levels of total neutrophil recruitment to lungs (~15 neutrophils/field of view), a significantly elevated number of streptavidin^+^ neutrophils was detected in lungs of mice subjected to cremaster muscle local hyperpermeability (Figure 9C). Furthermore, these studies indicated a significant association between numbers of streptavidin^+^ neutrophils and the extent of increased lung vascular permeability (Figure 9D) and a significant association between the presence of streptavidin^+^ neutrophils and sites of pulmonary vascular leakage (Figure 9, E and F). Collectively, the findings demonstrate that a local hyperpermeability reaction can promote distal organ injury by recruitment of rTEM neutrophils (Figure 10).

## Discussion

Enhanced microvascular leakage and neutrophil trafficking are pivotal features of an acute inflammatory response. Importantly, the molecular bases of these events are distinct (9) and there is ample evidence to show that neutrophil extravasation in vivo per se is not sufficient for increased microvascular leakage (present study and refs. 10, 11). Nonetheless, neutrophils, most notably when stimulated to adhere by certain chemoattractant mediators (27, 28), can secrete a range of permeability-enhancing factors (e.g., VEGF, LTA_4_, HBP, TNF) (12, 13, 29–31). Here we report for the first time to our knowledge that increased vascular permeability can also impact neutrophil trafficking by reversing the directional luminal-to-abluminal migration of neutrophils through the endothelium. Mechanistically, this was governed by disrupted localization of tissue chemokines as induced by the movement of chemokines into the vascular lumen through leaky venular walls. Crucially, we demonstrate that neutrophils stemming from hyperpermeability sites reenter the systemic circulation, exhibit an activated phenotype, and traffic to the lungs where they are present at sites of vascular injury. Collectively, through identifying a previously unreported link between two fundamental components of inflammation, our findings extend current understanding of pathological inflammation and suggest that targeting local microvascular permeability may provide an effective means of suppressing neutrophil-mediated remote organ damage.

Intrigued by the lack of investigations into the potential impact of increased vascular permeability on neutrophil trafficking, we applied high-resolution confocal IVM for simultaneous analysis of these phenomena. Our findings revealed that induction of vascular leakage does not grossly alter total tissue infiltration of neutrophils over several hours. However, unexpectedly, augmented vascular leakage rapidly promotes a significant frequency of neutrophils that have initiated TEM to exhibit retrograde motility within EC junctions and eventually reenter the blood circulation. Increased microvascular permeability consistently preceded the occurrence of this aberrant TEM response, suggesting a causal link. Direct evidence for this notion was acquired through the use of knockin mice expressing a Y685F mutant of VE-cadherin with selective impaired vascular permeability induction (9) that showed reduced disrupted neutrophil TEM. Neutrophil reverse migration within EC junctions, termed “reverse TEM,” has previously been described by our group in relation to multiple inflammatory scenarios in the murine microcirculation (6, 14, 15). This response, which was first described in vitro for human neutrophils (32) is, however, one of a number of neutrophil reverse migration modes that to date have been reported in numerous contexts, experimental models, and inflammatory conditions (23–25, 33–35). The wide-ranging profiles and potential implications of neutrophil retrograde migration begs the need for further explorations of this enigmatic response in terms of its mechanisms and consequences.

In addressing the mechanisms that drive neutrophil rTEM, our previous works identified NE-mediated cleavage of EC junctional JAM-C as a trigger of this cellular response (14, 15). Together with in vitro studies of monocyte TEM (36), these findings indicated a need for EC JAM-C as a regulator of 1-way leukocyte trafficking through EC junctions, although the precise molecular basis of JAM-C–mediated luminal-to-abluminal neutrophil motility remains unclear. Of note, however, directly acting vascular permeability–enhancing agents (histamine and VEGF) that effectively instigated neutrophil rTEM in IL-1β–stimulated tissues had no impact on JAM-C expression. As an alternative mechanism, and based on recent findings showing the significance of compartmentalized directional cues in promoting neutrophil diapedesis (6), we hypothesized that increased vascular permeability may disrupt the correct positioning of chemotactic signals within the venular wall niche. Focusing on CXCL1, a potent neutrophil chemoattractant abundantly generated within our acute inflammatory models, elevated plasma levels of this chemokine were noted in all hyperpermeability reactions tested. Intriguingly, these results suggested that vascular leakage can prompt the diffusion of endogenously generated CXCL1 from the tissue and/or the venular wall into the vascular lumen. Support for this notion was acquired through tracking of topically applied 10-kDa dextran in histamine-stimulated tissues by confocal IVM and such an event was predicted by mathematical modeling. Briefly, although under normal homeostatic conditions intact EC contacts provide a significant barrier to movement of molecules into tissues, this situation changes in inflammation. When vascular permeability is increased, EC junctional contacts loosen, and although this supports increased hydraulic flux, it also causes a decrease in hydraulic velocity. Such a scenario can facilitate diffusion of a small molecule (e.g., a chemokine) from the tissue back into the bloodstream as shown here both experimentally and mathematically. This hypothesis was conclusively validated through assessing the interstitial-to-vascular lumen distribution of exogenous hCXCL8 following specific genetic or pharmacological modulations of vascular permeability induction. Collectively, the findings reveal that hyperpermeability-enhancing inflammatory conditions can promote reverse diffusion of chemokines from the tissue to the vascular lumen, a phenomenon that can disrupt the directional motility of neutrophils through EC junctions. The latter was definitively illustrated in experiments where systemic blockade of CXCL1 prevented neutrophil rTEM in hyperpermeability reactions, and conversely, intravenous exogenous CXCL1 promoted neutrophil rTEM.

The sequence of molecular and cellular events that guide neutrophils from the vascular lumen to the interstitial tissue is well established and described by the leukocyte adhesion cascade (3). Here, it is considered that chemokines immobilized on the luminal aspect of blood vessels trigger the local arrest of neutrophils (3) with sequential, compartmentalized, and locally presented chemotactic cues within venular walls promoting luminal-to-abluminal diapedesis (6). Although glycosaminoglycans (GAGs) are considered to provide the principal mode of retaining chemokines on the luminal aspect of blood vessels, the retention of chemokines within EC junctions is likely mediated by binding to the atypical chemokine receptor ACKR1 that is enriched at these sites (6, 37). Together, owing to their pivotal role in localization, retention, and/or transport of chemokines, ACKR1 and GAGs are key molecular players in supporting leukocyte trafficking (38, 39). In contrast to these physiological regulatory modes, increased vascular permeability appears to account for excessive EC junctional motility and resultant entry of chemokines into the bloodstream. As such, local hyperpermeability disrupts the correct spatiotemporal presentation of chemotactic cues within venular walls, and in doing so, disrupts a phenomenon that is critical for efficient and unidirectional luminal-to-abluminal migration of neutrophils (6).

Although migration away from sites of inflammation and injury is now an established neutrophil behavior (34), it is highly plausible that the implications of this response are different in diverse contexts and in varied experimental models. Most notably, neutrophil retrograde motility within interstitial tissues away from sites of sterile injury, and in some cases reentry into the blood circulation, is proposed as a component of inflammation resolution (24, 33, 40, 41). For example, Wang and colleagues reported that neutrophils recruited to a murine thermal hepatic injury model contributed to revascularization of injured tissues and subsequently left the injured site by reentering the local vasculature (24). Using an elegant mouse model that selectively expressed a photoactivatable GFP in neutrophils, the authors could track approximately 10% of the tissue infiltrated neutrophils, a procedure that enabled a small number of cells to be tracked to the lungs and a higher number to the bone marrow. Phenotypic analysis of photoactivated neutrophils in these organs indicated increased expression of CXCR4. Based on the latter, it was concluded that neutrophil migration away from injured tissues is a physiological process that may regulate deactivation and/or reprogramming of neutrophils in the lungs before they are recruited to the bone marrow via CXCR4 for eventual clearance by apoptosis (24). The neutrophil reverse motility response reported in our study is distinctly different from that analyzed by Wang and colleagues in that it is restricted to EC junctions and the sub-EC space and collectively describes the reentry of transmigrating neutrophils back into the vascular compartment before the cells fully exit the venular wall. Although we have previously associated this response with remote organ damage (14, 15), our earlier works did not provide direct evidence for rTEM neutrophils trafficking to secondary organs. To address this vital point, here we have developed an in vivo cell-labeling method that precisely and efficiently distinguishes luminal neutrophils from all cells that breach EC junctions and hence tags all TEM and rTEM neutrophils stemming from the tissue under investigation. As such, a notable strength of our method is that it enables analysis of the full subpopulation of rTEM neutrophils migrating away from an inflammatory site in terms of their fate, phenotype, and pathophysiological relevance.

The application of our tracking method indicated dissemination of labeled rTEM neutrophils from a local hyperpermeability site to the systemic circulation where they showed an activated phenotype (increased CD11b and ICAM-1). In the same animals, labeled rTEM neutrophils were detected at significantly elevated levels in the pulmonary vasculature with an even greater activation state, exhibiting further increased expression of CD11b and ICAM-1, as well as β1 integrins and NE. The latter is highly indicative of degranulation contributing to some of the noted rTEM-neutrophil phenotypes and indeed neutrophils are known to express preformed stores of β2 and β1 integrins that can be mobilized to the cell surface during TEM (42). Certainly, there is ample evidence showing that engagement of neutrophils with EC junctional molecules can trigger signaling and transcriptional events within migrating leukocytes (42), suggesting that rTEM and tissue-infiltrated neutrophils may exhibit similar phenotypes. In line with this notion, although increased expression of ICAM-1 on neutrophils is a slow and transcriptionally regulated process (43), both tissue-infiltrated and rTEM neutrophils can exhibit an ICAM-1^hi^ phenotype (14, 32, 43). Of importance, however, while increased expression of integrins, other granular proteins, and ICAM-1 on tissue-infiltrated neutrophils collectively support effective breaching of venular walls, interstitial tissue migration, and pathogen clearance (42–46), such a phenotype on neutrophils that reenter the blood circulation could be highly detrimental to the host. In line with this notion, we detected a direct association between labeled rTEM neutrophils and lung injury, results that support the paradigm that rTEM neutrophils are a subset of activated neutrophils that can contribute to turning a local inflammatory response into a systemic phenomenon. The precise mechanism through which rTEM neutrophils exert tissue damage remains to be elucidated. However, the strong association of NE with the pathogenesis of numerous acute and chronic lung disorders (47) suggests that increased expression of this serine protease on rTEM neutrophils could be a significant factor. Furthermore, elevated expression of integrins, together with the ICAM-1^hi^ phenotype, may support increased aggregation and activation of rTEM neutrophils (e.g., degranulation and ROS generation) within small blood vessels of remote organs. Of direct relevance to this notion, we have previously shown that neutrophil ICAM-1 expression correlates with increased ROS generation (43). Additionally, pulmonary vasculature (but not blood) rTEM neutrophils showed increased expression of CXCR4, and rTEM neutrophils could be detected in the bone marrow 4 hours after their induction. These results collectively support the belief that neutrophils are retained within the lung vasculature in a transient manner during which they may be reprogrammed for homing to the bone marrow (24). The difference between our findings and those of other groups in terms of linking neutrophil rTEM to distant organ damage could lie in the nature of the reactions investigated, the type of retrograde migration being analyzed, and phenotype of the neutrophils stemming from the primary inflammatory site. Significant to our findings, acute lung injury is a life-threatening consequence of numerous local hyperpermeability-inducing conditions such as trauma, and pathologies induced by IR injury (48, 49). Thus, we propose that activated rTEM neutrophils stemming from local hyperpermeability inflammatory sites could provide a detrimental cellular link between primary and secondary sites of pathological inflammation.

In summary, the present results offer a causal link between increased local microvascular leakage and neutrophil rTEM, an axis associated with development of remote organ damage. Fundamental to this cascade of events is the discovery that increased vascular leakage can induce rapid translocation of chemokines from the interstitium into the systemic circulation against hydraulic flow, resulting in disrupted directional gradient across the venular wall. This response in turn drives the reentry of an activated subset of neutrophils back into the vascular lumen that can then contribute to development of lung injury. Collectively, our findings suggest that targeting excessive local microvascular permeability may be a plausible therapeutic strategy for protecting the host from secondary organ damage. Furthermore, our results could have implications for tumor cell intravasation and dissemination from primary sites of tumor growth that are characterized by leakiness of their blood vessels.

## Methods

### Abs.

The following Abs were obtained commercially: anti-CD31 (clone 390), PE–anti-CXCR4 (clone 2B11), anti–VE-cadherin (clone BV13), control anti-rabbit, anti–VE-cadherin (clone eBioBV14) mAbs from Thermo Fisher Scientific; PB–anti–Gr-1 (clone RB6-8C5), BV605–anti-CD62L (clone MEL-14), BV711–anti-CD11b (clone M1/70), AF488–anti-CD115 (clone AFS98), AF488–anti-CD102 (clone 3C4 MIC2/4), PE/Dazzle594–anti-CD54 (clone YN1/1.7.4), PE/Cy7–anti-CD29 (clone HMβ1-1), APC-Cy7–anti-CD115 (clone AFS98), biotin–anti–Ly-6G (clone 1A8) mAbs from BioLegend; blocking anti-CXCL1 (catalog 48415) from R&D Systems; rabbit anti–human CXCL8 (catalog NBP2-33819) from NOVUS; anti-NE (catalog ab68672) from Abcam; anti–α-SMA (clone 1A4), anti-CD31 (clone 2H8) from Sigma-Aldrich. Rabbit polyclonal anti–VE-PTP was generated as previously described (50). The following were gifts: rabbit polyclonal anti–JAM-C (clone H33; Michel Aurrand-Lions, INSERM, CRCM, France) and anti-MRP14 mAb (Nancy Hogg, Francis Crick Institute, London, United Kingdom).

### Animals.

Male WT C57BL/6 (Charles River) and *LysM-EGFP-ki* (51) mice (8–12 weeks old) were used for all studies. *VEC-Y685F-ki* mutant mice that exhibit a single point mutation in VE-cadherin were generated as previously described (9).

### Generation of chimeric mice.

*VEC-WT* and *VEC-Y685F* mice were lethally irradiated with 1 dose of 9 Gy over a time period of 11 minutes and were subsequently injected i.v. with 1.5 × 10^6^ to 3 × 10^6^ bone marrow cells from *LysM-EGFP-ki* mice. Level of engraftment was evaluated 4 weeks after reconstitution and all mice showed more than 99% of GFP^+^ neutrophils with similar neutrophil counts.

### Confocal IVM of the mouse cremaster muscle.

Anesthetized (3% isoflurane) male mice received an intrascrotal (i.s.) injection of fluorescently labeled anti-CD31 mAb (4 μg) and/or IL-1β (50 ng, R&D Systems), LTB_4_ (300 ng, Cambridge Bioscience) to label vessels within the tissue and/or induce an inflammatory response, respectively. Control animals received PBS. The cremaster muscles were then prepared for intravital imaging 2 hours or 30 minutes after IL-1β or LTB_4_ administration, respectively, as described previously (14, 15). Topical application of histamine (30 μM, Sigma-Aldrich) onto exteriorized tissues, or i.v. injection of VEGF (4 μg/mouse, R&D Systems) was used to induce vascular leakage. IR injury was induced as described previously (14, 15). In some experiments, anti-CXCL1 mAb or control IgG2a (1 mg/kg) and anti–VE-PTP Ab (100–200 μg) or rabbit IgG control (200 μg) was injected i.v. as indicated in relevant texts. In some experiments, recombinant mCXCL1 (50 ng, Peprotech) was injected i.v. 2 hours after IL-1β. To visualize vascular leakage, 75-kDa TRITC-dextran (40 mg/kg, Sigma-Aldrich) was injected i.v. (via tail vein cannula) 1 minute before the superfusion of histamine, in combination with i.v. injection of VEGF or during the reperfusion phase of IR-stimulated tissues. In some experiments, 10-kDa AF488-dextran (10 μg/mL, Thermo Fisher Scientific) was superfused 2 minutes after i.v. injection of 75-kDa TRITC-dextran for 10 minutes. The superfusate was then replaced with either Tyrode’s solution containing histamine (30 μM) or vehicle control for an additional 30 minutes. To label rTEM neutrophils, biotinylated anti-Ly6G mAb (2 μg) was injected i.v. 1.5 hours after IL-1β stimulation of tissues. Following exteriorization, the cremaster muscle was superfused with AF647-streptavidin (1 μg/mL, Thermo Fisher Scientific) with Tyrode’s solution with or without histamine (30 μM). *Z*-stack images of postcapillary venules (20–40 μm in diameter) were captured using a Leica SP5 or SP8 confocal microscope incorporating a ×20 water-dipping objective (NA 1.0), as detailed previously (6, 14).

### Quantification of neutrophil TEM, microvascular leakage, and streptavidin labeling.

Still images and 4D live recordings were analyzed using IMARIS software (Bitplane). Extravascular neutrophils were defined as those that had fully transmigrated and passed through the pericyte layer, recognizable via a change in their morphology, and expressed as number of cells/mm^3^ of tissue. rTEM neutrophils were defined as cells that moved in an abluminal-to-luminal direction within EC junctions. Normal neutrophil TEM was classified as a response in which the cells migrated through EC junctions only in a luminal-to-abluminal direction, as previously described (14, 15). Extravascular leakage was quantified by interstitial accumulation of i.v. 75-kDa TRITC-dextran and presented as mean fluorescence intensity (MFI) of indicated time points, measuring 6 to 8 regions of interest (ROI) in the interstitium (excluding areas exhibiting dextran-positive perivascular cells) and 30 μm away from the vessel wall. These readings were then normalized in relation to the first 2 MFI readings obtained after i.v. injection of the tracer and presented as normalized MFI. Similar analysis was conducted for the quantification of tissue levels of 10-kDa AF488-dextran (MFI normalized to the first 2 readings after superfusion of the tracer). Interstitial neutrophil speed and fluorescence intensity of AF647-streptavidin were analyzed using the spot or isosurface functions of IMARIS software, respectively.

### Bright-field IVM of the mouse cremaster muscle.

Mice were injected i.s. with IL-1β (50 ng) or PBS alone for 2 hours before cremaster muscle exteriorization and before the superfusion of histamine (30 μM) or vehicle, as described above. Leukocyte rolling, firm arrest, and extravasation within 20- to 40-μm postcapillary venules were quantified by IVM over a 1.5-hour period using a bright-light microscope (Axioskop FS, Carl Zeiss), as detailed previously (44). Several vessel segments (*n* = 3–5) from multiple vessels (*n* = 3–5) were quantified for each animal.

### Quantification of chemokine and dextran levels in tissue and plasma.

Anesthetized (3% isoflurane) mice were subjected to cremasteric ischemia (40 minutes) or injected i.s. with IL-1β (50 ng) or TNF (300 ng R&D Systems) in 200 μL PBS. Control animals received PBS only. Two hours later, animals were injected with i.s. histamine (30 μM solution) or PBS (both in 200 μL), or i.v. VEGF (4 μg/mouse) or PBS. Cremaster muscles and plasma samples (in 50 mM EDTA) were harvested 30 minutes later. In some experiments, hCXCL8 (500 ng, Peprotech) was coinjected with IL-1β (50 ng) for 1 hour before the end of the in vivo test period (i.e., 2.5 hours). In some experiments, mice were treated i.v. with a blocking anti–VE-PTP Ab (200 μg) (20, 21) or rabbit IgG control (200 μg) 30 minutes after hCXCL8 and IL-1β injection. Other experiments involved treating the mice with a blocking anti–VE-cadherin mAb (BV13; 100 μg) (22) or a control mAb (anti-CD31 mAb, clone 390; 100 μg) i.s. for 3 hours followed by local injection of hCXCL8 for 1 hour before tissue and plasma collection. Tissues were homogenized in PBS containing 0.1% Triton X-100 and 1% Halt Protease and Phosphatase Inhibitor cocktail (Thermo Fisher Scientific) and mechanically dissociated using the Precellys 24 bead-beating system (Bertin Technologies). Levels of mCXCL1 and hCXCL8 were analyzed as per the manufacturer’s instructions by ELISA (R&D Systems and Thermo Fisher Scientific, respectively: sensitivity, 2 pg/mL). The quantity of chemokine detected in tissues was normalized to protein content as determined using a BCA assay (Thermo Fisher Scientific). Levels of 10-kDa AF488-dextran in plasma was quantified using a BMG NOVOstar microplate reader (BMG LABTECH).

### Immunofluorescence staining and confocal analysis of tissues.

Whole-mount cremaster muscles were analyzed for expression of hCXCL8, JAM-C, red-(580/605)-microbeads (20 nm in diameter, 9.1 × 10^13^ beads; Thermo Fisher Scientific), and AF647-streptavidin as previously described (6, 15). Briefly, surgically removed tissues were fixed in ice-cold paraformaldehyde (PFA) (4% in PBS) for 45 minutes, blocked, and permeabilized at room temperature for 4 hours in PBS containing 25% FCS and 0.5% Triton X-100, and incubated overnight (anti-hCXCL8, anti-MRP14, anti–α-SMA, anti-CD31 staining) or 72 hours (anti–JAM-C staining) at 4°C with primary antibodies. Immunostained tissues were imaged with an inverted Zeiss 800 confocal laser-scanning microscope. JAM-C expression within the VE-cadherin channel was quantified as previously described (14, 15). For analysis of hCXCL8 localization, ECs, neutrophils, and pericyte isosurfaces were created based on regions immunostained for CD31 (CD31^hi^ junctional and CD31^dim^ nonjunctional regions), MRP14, and α-SMA, respectively. EC body and junctional hCXCL8 expression was quantified as MFI within these isosurfaced regions. The MFI of fluorescent beads present in the subendothelial space (<1 μm from ECs) was quantified as a measure of cremaster muscle vascular leakage. An isosurface using the CD31 channel was generated to exclude fluorescent signal of beads inside the vascular lumen. All protein expression levels were quantified from 6 to 12 images/tissue and expressed as MFI values of tissues stained with specific antibodies after subtraction of MFI values acquired from tissues stained with isotype control antibodies.

### Analysis of blood, pulmonary vascular washout, and bone marrow neutrophils by flow cytometry.

Mice received an i.v. injection of biotin–anti-Ly6G mAb (2 μg) 90 minutes after stimulation of cremaster muscles with locally administered IL-1β (50 ng) or PBS. Mice were locally injected 30 minutes later with histamine (200 μL of 30 μM solution) or PBS in combination with AF647-streptavidin (400 ng). Whole blood and lung vascular washout were collected 120 to 240 minutes later as previously described (14, 15), a method that recovered approximately 50% of the lung pulmonary vascular neutrophils. Bone marrow was isolated from 1 femur/animal. The samples were then analyzed using an LSR Fortessa flow cytometer (Becton Dickinson) and FlowJo software (Tree Star). Following doublets exclusion, neutrophils from *LysM-EGFP-ki* and WT mice were gated as LysM-EGFP^hi^GR-1^hi^ or GR-1^hi^CD115^–^, respectively. Leukocyte numbers were determined using fluorescent counting beads.

### Analysis of lung vascular leakage.

Pulmonary vascular leakage was quantified by adapting published methods (26). Briefly, mice were injected i.v. with crimson-(625/645)-microbeads (20 nm in diameter, 9.1 × 10^13^ beads; Thermo Fisher Scientific), biotin–anti-Ly6G (2 μg), and AF555–anti-CD31 mAb (10 μg) in 150 μL sterile PBS, 90 minutes after IL-1β stimulation of cremaster muscles (50 ng in 200 μL of PBS). Thirty minutes later, mice were injected i.s. with histamine (200 μL of 30 μM solution) or PBS in combination with Atto425-streptavidin (400 ng, Sigma-Aldrich) for 120 minutes. At the end of the reaction, mice were sacrificed, the descending vena cava was clamped, and ice-cold PFA (2% in PBS) was perfused via the right ventricle. Lung lobes were excised and placed on top of a cover slip and were imaged immediately in situ with an inverted Zeiss 800 confocal laser-scanning microscope. The MFI of fluorescent beads present in the extravascular space (5–7 images/tissue from different lung lobes) was quantified as a measure of lung vascular leakage. For this purpose, an isosurface was generated using the anti-CD31 mAb to exclude fluorescent signal of beads inside the vascular lumen. To quantitatively analyze the association between streptavidin^+^ neutrophils and regions of vascular leakage, we arbitrarily defined streptavidin^+^ neutrophil regions as areas that expressed at least 1 streptavidin^+^ neutrophil within a perimeter of 60 μm. Control areas within the same image were defined as similarly sized regions that were devoid of streptavidin^+^ neutrophils.

### Statistics.

Data analysis was performed using Prism 6 (GraphPad Software). Results are expressed as mean ± SEM and the *n* values for each data set are provided in the figure legends. Statistical significance was assessed by 2-tailed Student’s *t* test, or 1-way or 2-way ANOVA followed by Bonferroni’s post hoc test. A *P* value less than 0.05 was considered significant.

### Study approval.

All in vivo experiments were conducted under the United Kingdom legislation according to the Animal Scientific Procedures Act 1986, with all procedures being conducted in accordance with United Kingdom Home Office regulations.

## Author contributions

COW, RJ, MBV, and SN designed the experiments. COW and RJ did most of the experiments and compiled and analyzed the data. COW initiated the experimental work. AB and LR contributed to certain key experiments. COW and RJ prepared the figures. KPA and DOB conducted the mathematical modeling. BM, AFN, TG, MS, and MG were involved in specific experiments. DV provided resources and advised on experimental protocols. COW, RJ, MBV, and SN wrote the manuscript. MBV and SN funded the work and provided overall research supervision. SN conceived the project.

## Supplementary Material

Supplemental data

Supplemental Video 1

Supplemental Video 2

Supplemental Video 3

Supplemental Video 4

Supplemental Video 5

Supplemental Video 6

## Version 1 01/23/2020

In-Press Preview

## Version 2 03/23/2020

Electronic publication

## Version 3 05/01/2020

Print issue publication
