# Supplementary material for: Local microvascular leakage promotes trafficking of activated neutrophils to remote organs
Source: J Clin Invest. 2020 Mar 23;130(5):2301–18. doi: 10.1172/JCI133661 (PMC7190919; doi:10.1172/JCI133661)
Supplement: Supplemental data [file jci-130-133661-s052.pdf]

Supplemental material

Local microvascular leakage promotes trafficking of activated neutrophils to remote organs

Charlotte Owen-Woods[1], Régis Joulia[1], Anna Barkaway[1], Loic Rolas[1], Bin Ma[1], Astrid Fee Nottebaum[2], Kenton P Arkill[3], Monja Stein[1], Tamara Girbl[1], Matthew Golding[1], David O Bates[3], Dietmar Vestweber[2], Mathieu-Benoit Voisin[1] & Sussan Nourshargh[1,4*]

[1]William Harvey Research Institute, Barts and The London School of Medicine and Dentistry, Queen Mary University of London, Charterhouse Square, London, EC1M 6BQ, UK.

[2]Department of Vascular Cell Biology, Max Planck Institute for Molecular Biomedicine, Röntgenstraße 20, 48149 Münster, Germany.

[3]Division of Cancer and Stem Cells, School of Medicine, University of Nottingham Biodiscovery Institute, University Park, Science Road, Nottingham NG7 2RD UK

[4]Centre for Inflammation and Therapeutic Innovation, Barts and The London School of Medicine and Dentistry, Queen Mary University of London, London, EC1M 6BQ, UK.

C. Owen-Woods and R. Joulia contributed equally to this work.

A. Barkaway and L. Rolas contributed equally to this work.

M-B. Voisin and S. Nourshargh jointly supervised the work.

*Correspondence to Sussan Nourshargh: s.nourshargh@qmul.ac.uk

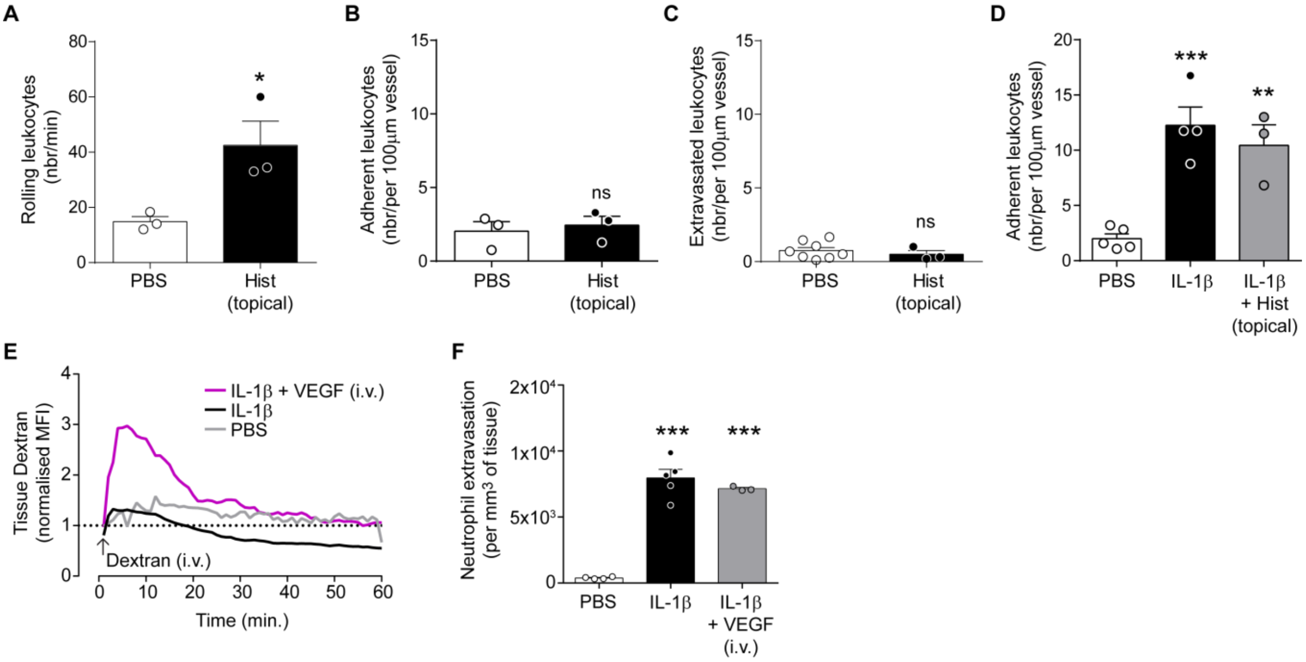

Supplemental Figure 1. Effect of topical histamine and systemic VEGF on neutrophil-vessel wall interactions and microvascular leakage in the cremaster muscle. (**A**-**C**) Cremaster muscles of WT mice were exteriorised, superfused with either histamine (30 μM) or vehicle (PBS) and analysed for neutrophil-vessel wall interactions by brightfield IVM. Number of rolling (**A**, cell number/min, n = 3 mice per group), adherent (**B**, n = 3 mice per group) and extravasated (**C**, n = 3-8 mice per group) leukocytes per 100 μm length of post-capillary venules. (**D**) Effect of topical histamine (30 μM) on leukocyte adhesion in IL-1β-stimulated tissues (n = 3-5 mice per group). (**E** and **F**) *LysM-EGFP-ki* mice, subjected to an i.s. injection of an anti-CD31 mAb to label endothelial cells junctions, were injected with i.s. PBS or IL-1β (50 ng) for 2 h. The mice were then injected i.v. with VEGF (4 μg/mouse) and 75 kDa-TRITC-dextran and neutrophil responses and vascular leakage were analysed by confocal IVM. (**E**) Time-course of dextran leakage into the perivascular region of a selected post-capillary venule (n = 3-5 mice per group). (**F**) Neutrophil extravasation (n = 3-5 mice per group). Data are represented as mean ± SEM (each dot represents one mouse and one independent experiment). Statistically significant differences from PBS are shown by *p<0.05, **p<0.01 ***p<0.001, two-tailed Student's t-test or one-way ANOVA followed by Bonferroni's post hoc test (ns = not significant).

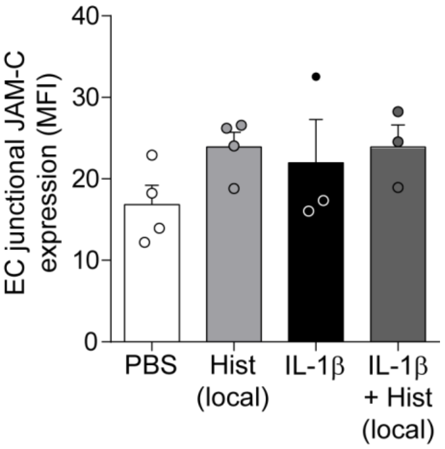

Supplemental Figure 2. Histamine has no impact on endothelial cell junctional JAM-C expression. Cremaster muscles of WT animals were injected with IL-1β or PBS injection (2 h) prior to the local administration of histamine or vehicle for 30 mins. Tissues were then collected, immunostained for JAM-C and the expression of EC junctional JAM-C was quantified by confocal microscopy (n = 3-4 mice per group). Data are represented as mean ± SEM (each dot represents one mouse and one independent experiment). No statistical differences were obtained between any of the groups and PBS as analysed using one-way ANOVA followed by Bonferroni's post hoc test.

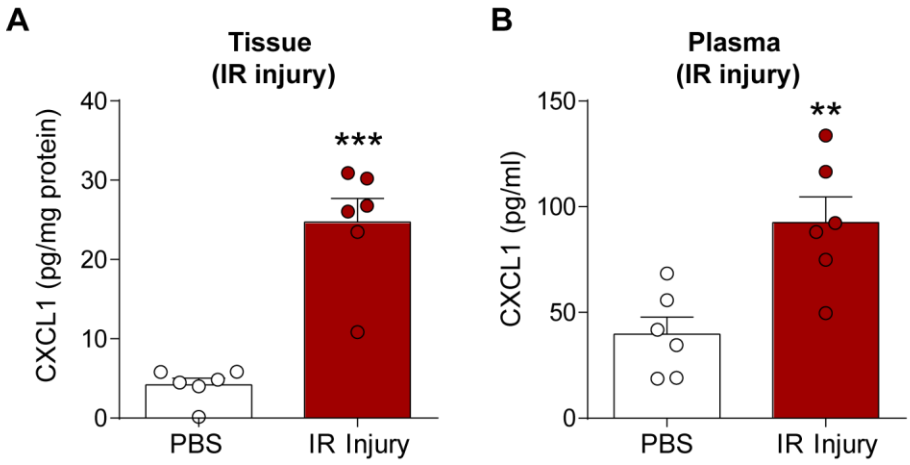

Supplemental Figure 3. Mice subjected to cremasteric IR injury exhibit elevated levels of CXCL1 in tissues and plasma. Cremaster muscles of WT animals were subjected to IR injury (40 min ischemia followed by 30 min reperfusion period). Tissue (**A**) and plasma (**B**) were collected and analysed for CXCL1 levels by ELISA (n = 6 mice per group). Data are represented as mean ± SEM (each dot represents one mouse and one independent experiment). Statistically significant differences from PBS are shown by **p<0.01, ***p<0.001, two-tailed Student's t-test.

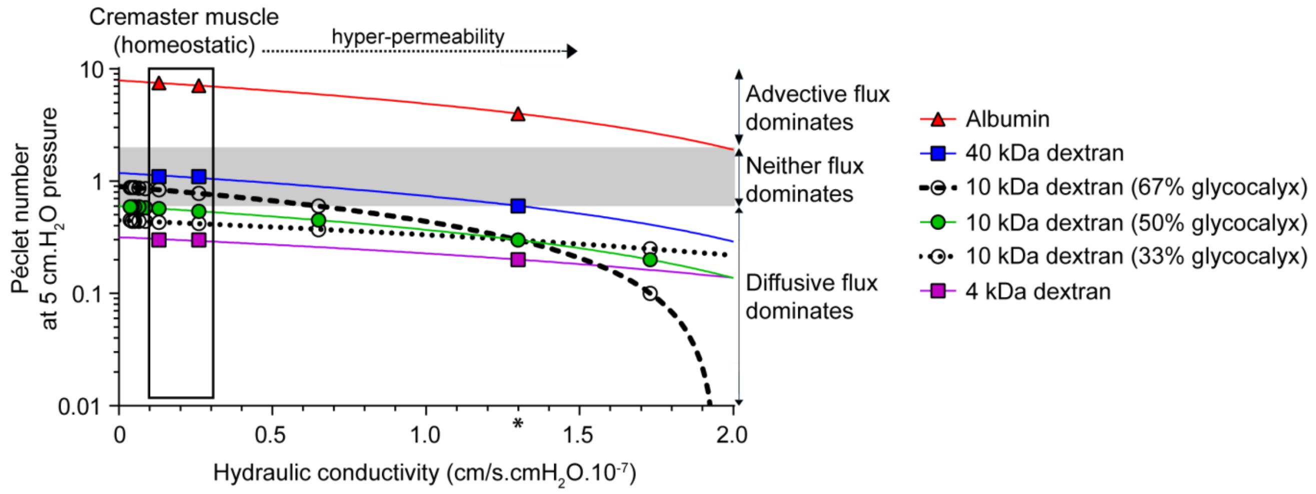

Supplemental Figure 4. Mathematical modelling of molecular flux across cremasteric venules. Estimation of the Péclet number at 5 cm.H_2O net pressure difference for 4 kDa dextran, 40 kDa dextran, albumin and 10 kDa dextran assuming different distributions of resistance across the vessel wall (67% of the resistance across the glycocalyx, 50% across the glycocalyx or 33% across the glycocalyx). The hydraulic conductivity is well characterised from published works as detailed below. The boxed data indicate the hydraulic conductivity in the cremaster muscle microcirculation under homeostatic conditions (0.1-0.3x10^{-7}cm/s.cmH_2O) with the arrow indicating changes in hydraulic conductivity following hyperpermeability.* Indicates typical value for more permeable vessels (e.g. mesenteric capillaries).

Text associated with Supplementary Figure 4

Molecular flux is determined by a combination of diffusive and advective transport. For a macromolecule produced extravascularly to enter the circulation, the diffusive flux from tissue to blood must be high enough to overcome the opposing advective flux. This ratio can be defined by the Péclet number (Pe):

$$Pe = \frac{Advective\ Transport\ Rate}{Diffusive\ Transport\ Rate} = \frac{V.L}{D} \qquad \text{EQ. 1}$$

The *advective transport rate* is the hydraulic velocity (*V*) and the *diffusive transport rate* is the diffusion coefficient (*D*) divided by the distance (*L*), determined in the present study by the endothelial intercellular cleft length (1). If the Pe is near to 1 then neither fluxes are dominant; if the Pe is much greater than 1 then diffusion cannot effectively oppose filtration and the molecule cannot travel against the flow; if Pe is much less than 1, then there can be diffusion against the flow of fluid. Although the exact combination and interaction of blood vessel wall molecular and cellular structures on venular wall Pe is unknown, particularly during inflammation, a solid understanding of the permeability of the

vessel wall as a whole to both macromolecules and to water (hydraulic conductivity) enables mathematical modelling of molecular flux across cremasteric venular walls. In normal physiology, in continuous vessels such as those in the muscle, the fluid from the pressurised small blood vessel (e.g. postcapillary venules) needs to move through the endothelial cell (EC) glycocalyx, funnelled through the EC intercellular clefts and then further funnelled through the breaks in junctional strands within the EC clefts. In our case *equation 1* can be rewritten for a molecule *i* where $L_p$is the hydraulic conductivity, L_{cleft} the cleft length, D^i_{Cleft} the diffusion coefficient in the cleft, ΔP the net change of pressure, and A_{Open} the area of open cleft available to transport per unit area.

$$Pe^i = \frac{\left[\frac{Lp}{\Delta P.A_{Open}}\right].L_{cleft}}{D^i_{Cleft}} \qquad \text{EQ. 2}$$

Due to the extensive body of research on mesenteric microcirculation permeability the following calculations will be based on mesenteric vessels and then adapted for the cremaster blood vessel where only L_p has been measured.

For every 1 µm^2 of blood vessel surface area there are 0.1 µm of EC intercellular cleft with approximately 0.01 µm of EC open junction for rat mesenteric vessels (2). This equates to 1000 cm of cleft for each cm^2 of venular wall, and 100 cm of open junction. The L_p in the mesenteric vessels is approximately 1.3x10^{-7} cm.s^{-1}.cmH_2O^{-1} and the net driving pressure (Hydrostatic minus Oncotic pressures) would be ~5 cmH_2O (2, 3). If the EC intercellular cleft is 18 nm wide then (maintaining traditional units) the fluid velocity though these slits (V) is calculated to be 3.6x10^{-3} cm/s. D^i_{Cleft} can be calculated through a slit (4, 5):

$$D^i_{Cleft} = D^i_{Free}[1 - 1.004B + 0.418B^3 + 0.210B^4 - 0.1696B^5] \qquad \text{EQ. 3}$$

Where:

$$B = 2a^i/W_{Cleft} \qquad \text{EQ. 4}$$

With a^i as the molecular radius and W_{Cleft} as the cleft width.

D^i_{Cleft} for a 10 kDa dextran in the cleft ($D^{10\,kDa\,dextran}_{Cleft}$) is calculated as 9.3x10^{-7} cm^2.s^{-1}, given an 18 nm slit, assuming free diffusion of 1.1x10^{-6} cm^2.s^{-1} (6) and a Stokes-Einstein radius of 2.3 nm (manufacturer datasheet). The estimated length of the cleft varies from 400 nm to nearer 1000 nm, but assuming a mean length of 700 nm (7) the estimate of $Pe^{10\,kDa\,dextran}$ is 0.3.

For comparison with albumin (using 3.5 nm for the radius (8) and 1.0x10^{-7} cm^2.s^{-1} for free diffusion), $Pe^{Albumin}$ = 4.

Cremaster muscle vessels have not to date been analysed to the same detail but the tissue has previously been estimated to have between 5x and 10x lower L_p (9, 10). These experiments also noted that limiting pore sizes were unlikely to be the reason for the difference between muscle and mesenteric blood vessels, and therefore the available proportion of EC surface area was given as the most likely explanation. Intuitively, if we have only 10% of the available EC cleft area, the resistance is 10-fold higher (L_p is reduced by 10-fold) and would not change the Pe^i. However, the resistance from the endothelial glycocalyx in the mesenteric vessels is similar in magnitude (50% to 150%) to the EC cleft resistance, and therefore contributes between 33 and 67% of the total resistance across the vessel wall. This means that the available area of the cleft would need to be reduced by more than 10-fold (15-fold to 30-fold respectively) to produce a 10-fold reduction in L_p and hence the Pe^i must be greater in the case of cremaster vessels. In Supplemental Figure 4, we model Pe for 10kDa dextran at different hydraulic conductivity and percent contribution of the glycocalyx to endothelial resistance. It can be seen that Pe^{10kDa} rises from 0.3 in the mesentery (L_p ~ $1.3x10^{-7}$ $cm.s^{-1}.cmH_2O^{-1}$) to between at least 0.4 and likely greater than 0.8 in the cremaster. Therefore the movement of a small molecular weight protein (e.g. a chemokine or 10 kDa dextran) back into the vasculature is likely to be finely balanced under normal conditions as the $Pe^{10kDa\ dextran}$ is approaching unity.

Calculation of Pe^{10kDa} during a hyperpermeability inflammatory response:

The experimental results in this manuscript (see Figure 4) show that topical application of 10 kDa dextran to exteriorised cremaster muscles led to detection of a low level of this molecule in the vasculature under homeostatic conditions. This is in line with the preceding mathematical expectations. Upon addition of histamine, higher levels of 10 kDa dextran were detected in the vasculature, i.e. a decrease in Pe^{10kDa} to substantively less than 1. Since the experiment also involved tracking i.v. injected 75 kDa dextran (used to represent an albumin sized molecule), which was correspondingly increased in the interstitium, these results indicate an increase in hydraulic flux. Clearly, any increase in water flux without a structural change in the cleft region would increase Pe^i rather than decrease it, strongly indicating that in these works a structural change to the vessel had been induced. The principal possibilities are changes to the endothelial glycocalyx or within the EC cleft region.

An endothelial glycocalyx with a 100 nm thick filtration zone exhibiting 9 nm pores with 20 nm spacing has much lower D^i_{Glx}, but as its available surface area is markedly greater covering the whole EC cleft length and the EC cleft entrance (circa 200 nm wide), it may impact the hydraulic velocity within each pore by reducing it (11, 12). Using the above model for diffusion across the EC glycocalyx (using the D^i_{Pore} versions of *Equation 3 and 4*), $Pe^{10\ kDa\ dextran}$ = 0.004 and 0.002 in cremaster and mesenteric vessels respectively ($Pe^{Albumin}$ = 0.1 and 1 respectively) and as such indicates that 10 kDa dextran is completely diffusion dominant in the endothelial glycocalyx. These calculations strongly indicate that

in our experimental model the transport of a 10 kDa molecule from the tissue to the lumen is defined by the EC cleft component of the vessel wall and under increased permeability

If histamine primarily changes the junctional strands in the cremaster exchange vessels, effectively increasing A_{Open} to that of the mesentery (or beyond), $Pe^{10\ kDa\ dextran}$ decreases from near unity to 0.3 (or further). This change results in the 10 kDa dextran diffusion flux to be dominant and hence for the molecule to be far more capable of travelling from the interstitium to the vasculature, even in the presence of the increased hydraulic flux from the vessel to the interstitium.

To comprehend the 75 kDa dextran (representing albumin sized molecules) flux increase one can use the effective solute permeability (P_S). This has been derived numerous times by Curry's group (13) and others, based on calculations by Patlak and colleagues (14)*:*

$$P_S = \left(\frac{e^{Pe}}{e^{Pe-1}}\right)\left[L_p(1-\sigma)\Delta P\right] \qquad \text{EQ. 5}$$

Where the σ is the reflection coefficient, for albumin $\sigma_{Albumin}$ = 0.95 (15). Utilising our parameters for the endothelial cell glycocalyx, the primary albumin resistance, the effective permeability rises from 3.3 x10^{-8} cm.s^{-1} to 5.0 x10^{-8} cm.s^{-1} for the transition from cremaster to mesenteric blood vessels. The tissue albumin concentration would therefore be expected to increase with the expected increase in junctional strand breaks, as observed by the increase in interstitial 75 kDa dextran. Of note is the sensitivity and dependence on Pe: increasing the ΔP by 5 cm.H_2O will make a 1.5-fold change to the mesenteric effective permeability but only 1.05-fold to the cremaster. Our estimate here is low compared to experimental data that ranges from 0.4 x10^{-6} cm.s^{-1} to 3 x10^{-6} cm.s^{-1} but these have various other caveats namely that ΔP was higher, dramatically increasing the sensitive parameter Pe. Further the mathematical derivation, although not phenomenological, is simplistic for several reasons, in particular a perfect membrane of zero thickness.

In conclusion, we have mathematically shown that in normal physiology, the diffusion of a 10 kDa molecule in the cremaster muscle tissue would be expected to be finely regulated. If the breaks in the EC junctional strands are more common, such as when induced by the inflammatory agent histamine that increases vascular permeability, then the tissue concentration of albumin sized molecules will be higher and at the same time, the 10 kDa molecule will be able to enter the vascular lumen. This mathematical prediction fits the experimental observations of the present study.

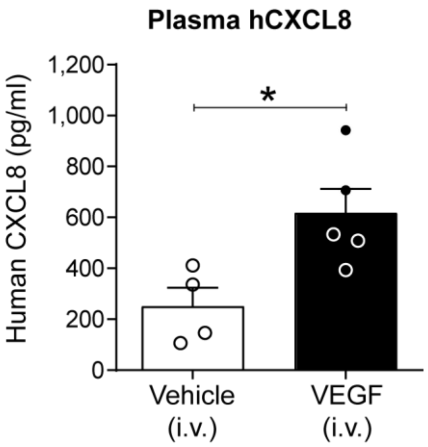

Supplemental Figure 5. VEGF promotes leakage of locally applied human CXCL8 from the tissue into the vascular compartment. Cremaster muscles were injected locally with IL-1β (50 ng) and human CXCL8 (hCXCL8, 500ng) for 1 h, followed by injection (i.v.) of vehicle (control) or VEGF (4 μg) for a further 1 h. hCXCL8 levels in plasma as quantified by ELISA (n = 4-5 mice per group). Data are represented as mean ± SEM (each dot represents one mouse and one independent experiment). Statistically significant difference from vehicle is indicated by *p<0.05, two-tailed Student's t-test.

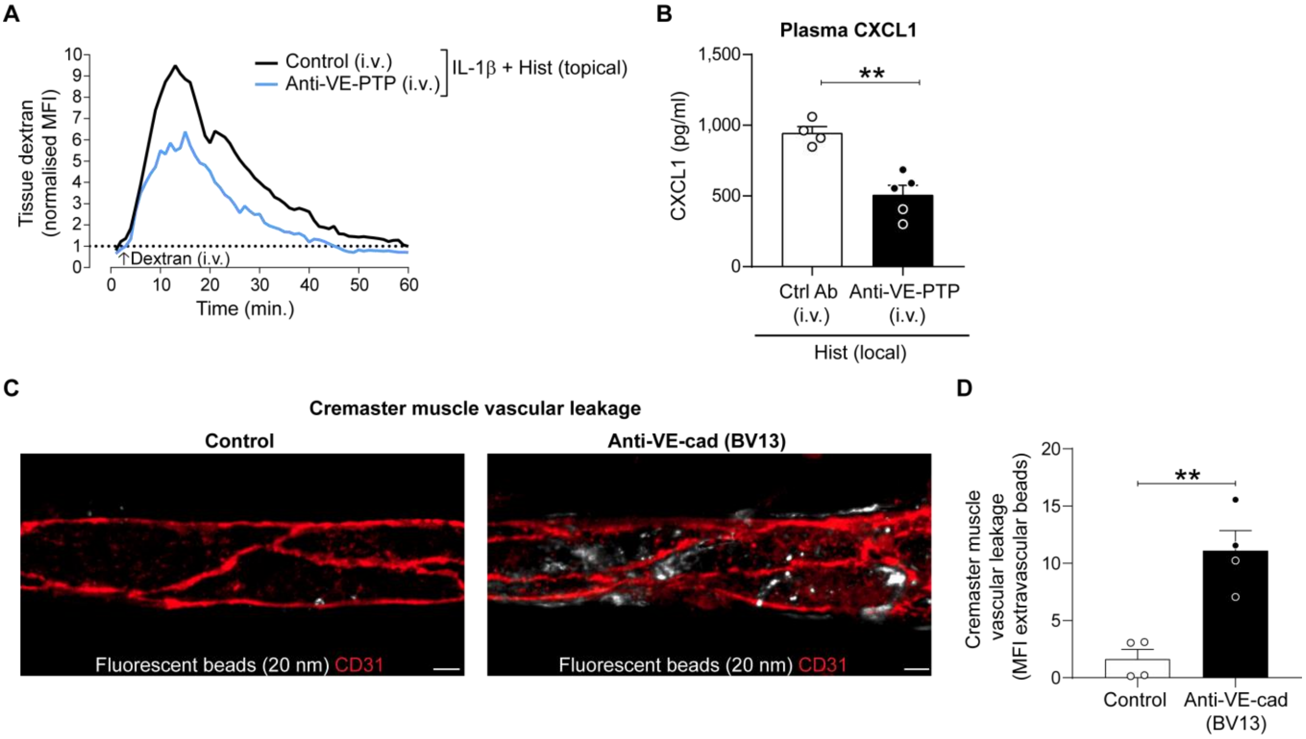

Supplemental Figure 6. Effect of blocking anti-VE-PTP and anti-VE-cadherin antibodies on vascular leakage and chemokine level. **(A-B)** Cremaster muscles of WT mice were stimulated with IL-1β (50 ng for 2 h) followed by topical superfusion of histamine onto exteriorised tissues. Blocking anti-VE-PTP mAb (100-200 μg) was injected i.v. 30 min before exteriorisation of tissues. Control mice received i.v. PBS or control rabbit IgG (no differences were observed between the 2 groups). Fluorescently-labelled anti-CD31 mAb was injected i.s. to visualise endothelial cell junctions and 75 kDa TRITC-dextran was injected i.v. at the beginning of the image acquisition period to visualise and quantify vascular leakage. (**A**) Time-course of dextran accumulation in the perivascular region of selected stimulated post-capillary venules. Tissue dextran accumulation is represented as normalised MFI (n = 3-4 mice per group). (**B**) CXCL1 levels in plasma as quantified by ELISA (n = 4-5 mice per group). (**C**-**D**) Cremaster muscles of WT mice were injected i.s. with a blocking anti-VE-cadherin mAb (BV13, 100 μg) for 4 h. Control mice were injected locally with PBS or anti-CD31 mAb (390, 100 μg) (data pooled as no difference was noted between the groups) for 4 h. Thirty minutes before the end of the in vivo test period, mice were injected i.v. with red-(580/605)-microspheres (20 nm in diameter, 9.1 x 10^{13} beads). Tissues were then collected and immunostained for anti-CD31 (clone 2H8, red) and analysed by confocal microscopy. (**C**) Representative confocal images of post-capillary venular segments from indicated reactions. Scale bars, 5 μm. (**D**) Cremaster muscle vascular leakage as quantified by accumulation (MFI) of extravascular beads (n = 4 mice per group). Statistically significant difference from control is indicated by **p<0.01, two-tailed Student's t-test.

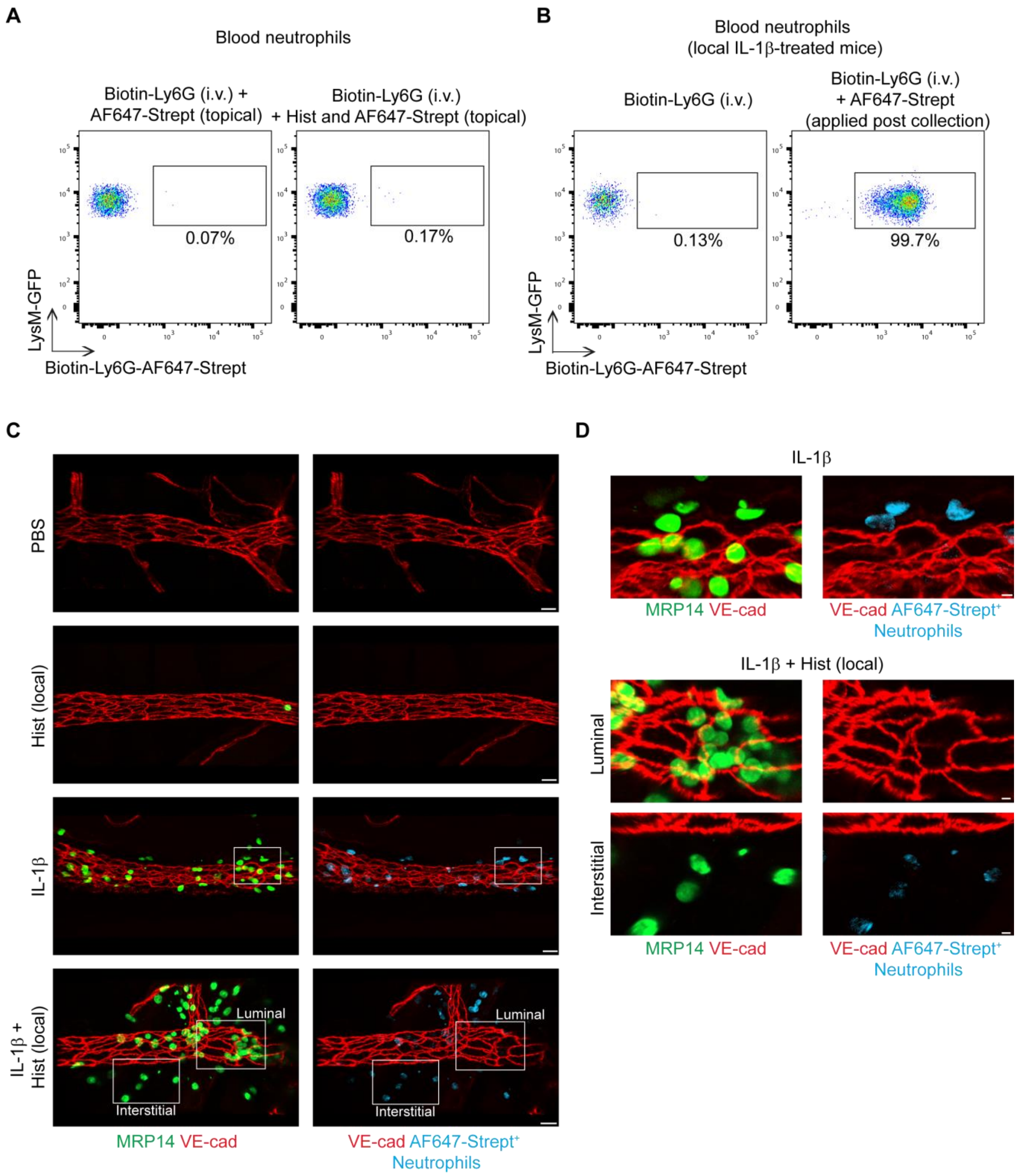

Supplemental Figure 7. Further characterisation of the biotin-Ly6G-AF647-streptavidin in vivo labelling method. (**A**) *LysM-EGFP-ki* mice received an i.v. injection of biotin-Ly6G mAb (2 μg) for 30 min, after which cremaster muscles were exteriorised and superfused with AF647-streptavidin (1 μg/ml) in combination with histamine or vehicle for 2 h. Peripheral blood was collected and analysed by FACS with representative flow cytometry profiles showing the percentage of AF647-Streptavidin^{+} neutrophils presented (representative of 3 independent experiments). (**B**) Cremaster muscles of

LysM-EGFP-ki mice were stimulated with IL-1β (50 ng for 2 h) and the mice were injected i.v. with biotin-anti-Ly6G mAb (2 μg) at t = 90 min. Blood was collected 2 h later and stained with AF647-strept in vitro to analyse the efficiency of biotinylated-anti-Ly6G labelling of circulating neutrophils. Representative flow cytometry profiles showing the percentage of AF647-Streptavidin$^+$ neutrophils (representative of 5 independent experiments). (**C** and **D**) Cremaster muscles of WT mice were stimulated locally with IL-1β (50 ng) or PBS for 2 h and the mice were injected i.v. with biotin-anti-Ly6G (2 μg) at t = 90 min. The mice were then injected either with i.s. histamine (200μl of 30 μM solution) or PBS all in conjunction with AF647-streptavidin (400 ng) for 2 h. Tissues were then collected and immunostained for VE-cadherin (red) and MRP14 (neutrophil, green) and analysed by confocal microscopy. (**C**) Representative confocal images of post-capillary venular segments from indicated reactions (left panels) and (**D**) enlarged images of boxed regions where AF647-streptavidin staining is shown within neutrophil isosurface masks (representative of 3 independent experiments). Scale bars, 15 μm (**C**) and 4 μm (**D**).

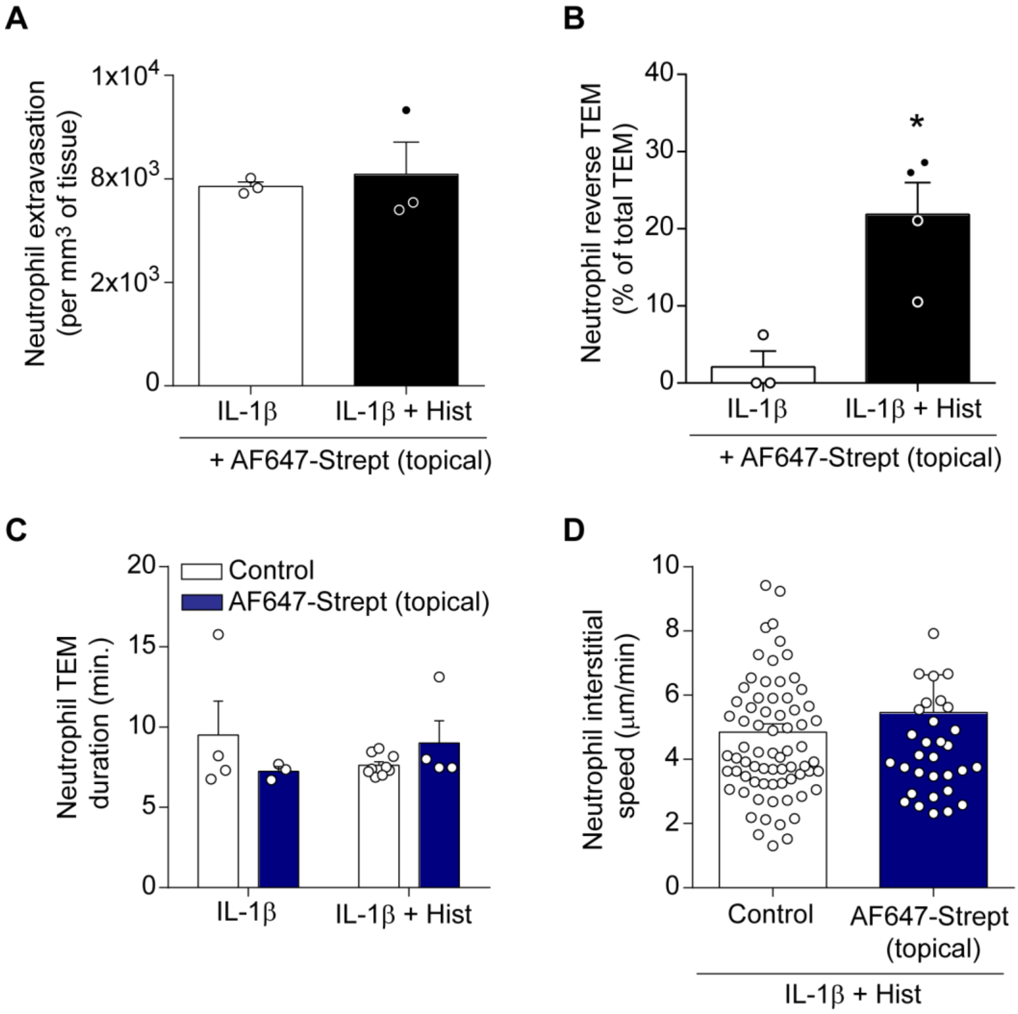

Supplemental Figure 8. The biotin-Ly6G-AF647-streptavidin labelling strategy has no impact on neutrophil motility in vivo. Cremaster muscles of *LysM-EGFP-ki* mice were stimulated locally with IL-1β (50 ng for 1.5 h) followed by an i.v. injection of biotin-anti-Ly6G mAb (2 µg) at t = 90 min. Tissues were then exteriorised and superfused with histamine (30 µM) or vehicle in combination with AF647-streptavidin (1 µg/ml) for 2 h. (**A**) Total neutrophil extravasation (n = 3 mice per group) and (**B**) frequency of neutrophil rTEM (n = 3-4 mice per group) show similar levels compared to reactions quantified in mice not subjected to the labelling strategy (see Figure 2A and D). (**C** and **D**) Mice subjected to the biotin-Ly6G-AF647-streptavidin labelling strategy and unlabelled mice exhibited similar neutrophil TEM duration (**C**, n = 3-9 mice per group) and neutrophil interstitial migration speed (**D**, n = 32-74 neutrophils per group) in cremaster muscles following local stimulation with IL-1β or IL-1β+histamine. Data are represented as mean ± SEM (each point represents one mouse and one independent experiment). Statistically significant differences from IL-1β are shown by *p<0.05, two-tailed Student's t-test.

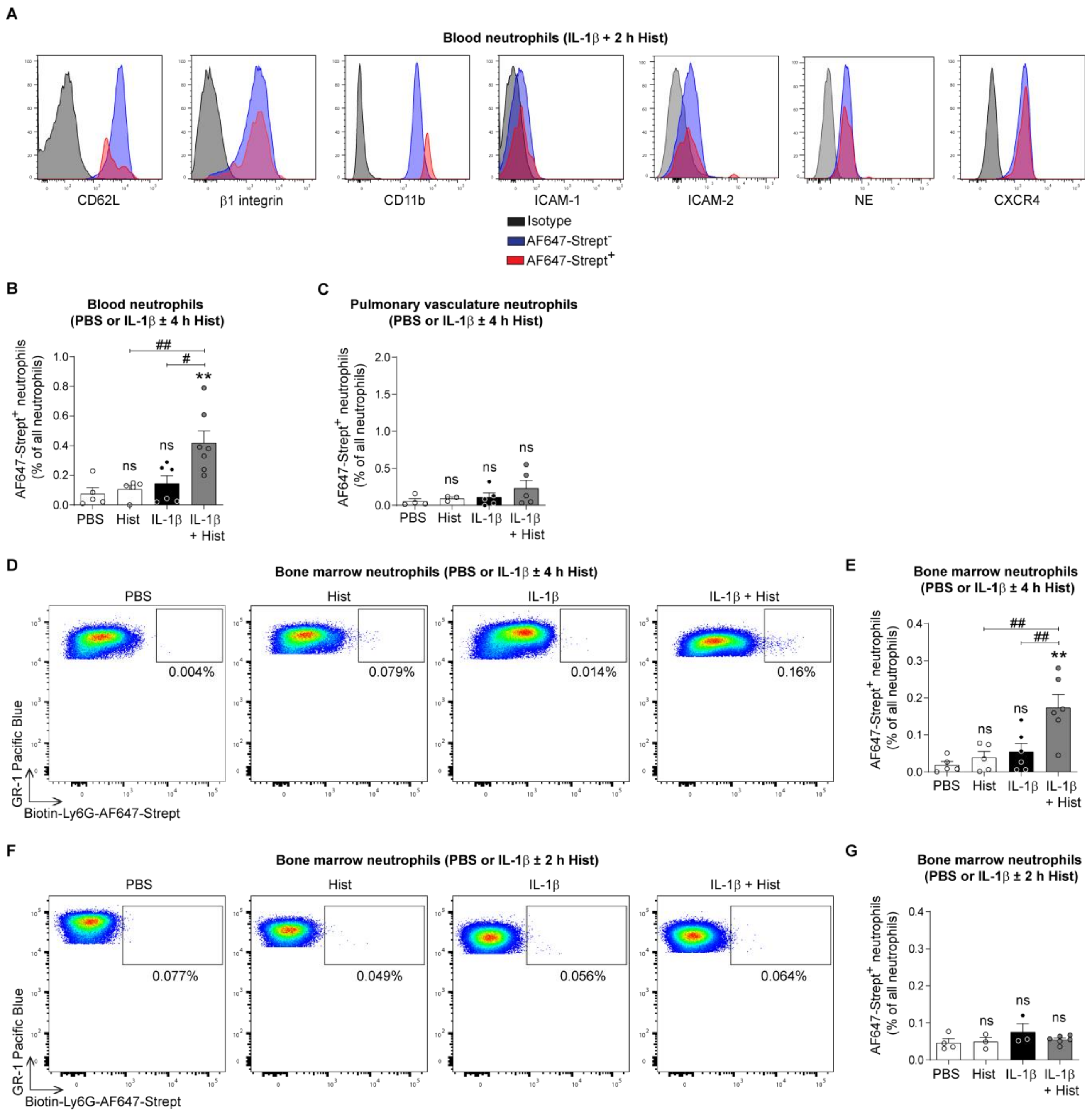

Supplemental Figure 9. In mice subjected to a local hyperpermeability reaction blood AF647-streptavidin$^+$ neutrophils show a distinct activated phenotype and streptavidin$^+$ neutrophils are present in the bone marrow at 4 h but not at 2 h post histamine. Cremaster muscles of WT mice were stimulated with IL-1β (50 ng) or PBS for 2 h followed by an i.s. injection of histamine (200 μl of 30 μM solution) or PBS. Mice were subjected to our biotin-Ly6G-AF647-streptavidin labelling strategy and as such received an i.v. injection of biotin-anti-Ly6G mAb (2 μg) at t = 90 min and were also injected locally with AF647-streptavidin (400 ng) in conjunction with histamine or vehicle for 2-4 h. Peripheral blood, pulmonary vascular washout and bone marrow samples were collected and analysed by flow cytometry. (**A**) Representative flow cytometry histograms of blood neutrophils from

a mouse subjected to cremaster muscle stimulation with IL-1β+histamine and biotin-Ly6G-AF647-streptavidin labelling strategy 2 h post histamine. The sample is assessed for the indicated markers and comparing AF647-Streptavidin^{+} neutrophils with AF647-Streptavidin^{-} neutrophils with relevant isotype control profiles being shown in black (representative of five independent experiments). (**B**) Frequency of blood AF647-Strept^{+} neutrophils (Gr-1^{high}/CD115^{-}) in mice subjected to the indicated cremasteric stimulations (n = 5-7 mice per group). (**C**) Frequency of pulmonary vascular washout AF647-Strept^{+} neutrophils (Gr-1^{high}/CD115^{-}) in mice subjected to the indicated cremasteric stimulations (n = 3-5 mice per group). (**D**) Representative flow cytometry profiles and (**E**) frequency of bone marrow AF647-Strept^{+} neutrophils (Gr-1^{high}/CD115^{-}) in mice subjected to the indicated cremasteric stimulations (n = 5-6 mice per group). (**F**) Representative flow cytometry profiles and (**G**) frequency of bone marrow AF647-Strept^{+} neutrophils (Gr-1^{high}/CD115^{-}) in mice subjected to the indicated cremasteric stimulations (n = 3-6 mice per group). Data are represented as mean ± SEM (each dot represents one mouse and one independent experiment). Statistically significant differences from PBS are shown by **p<0.01 or by indicated comparisons #p<0.05, ##p<0.01, one-way ANOVA followed by Bonferroni's post hoc test (ns = not significant).

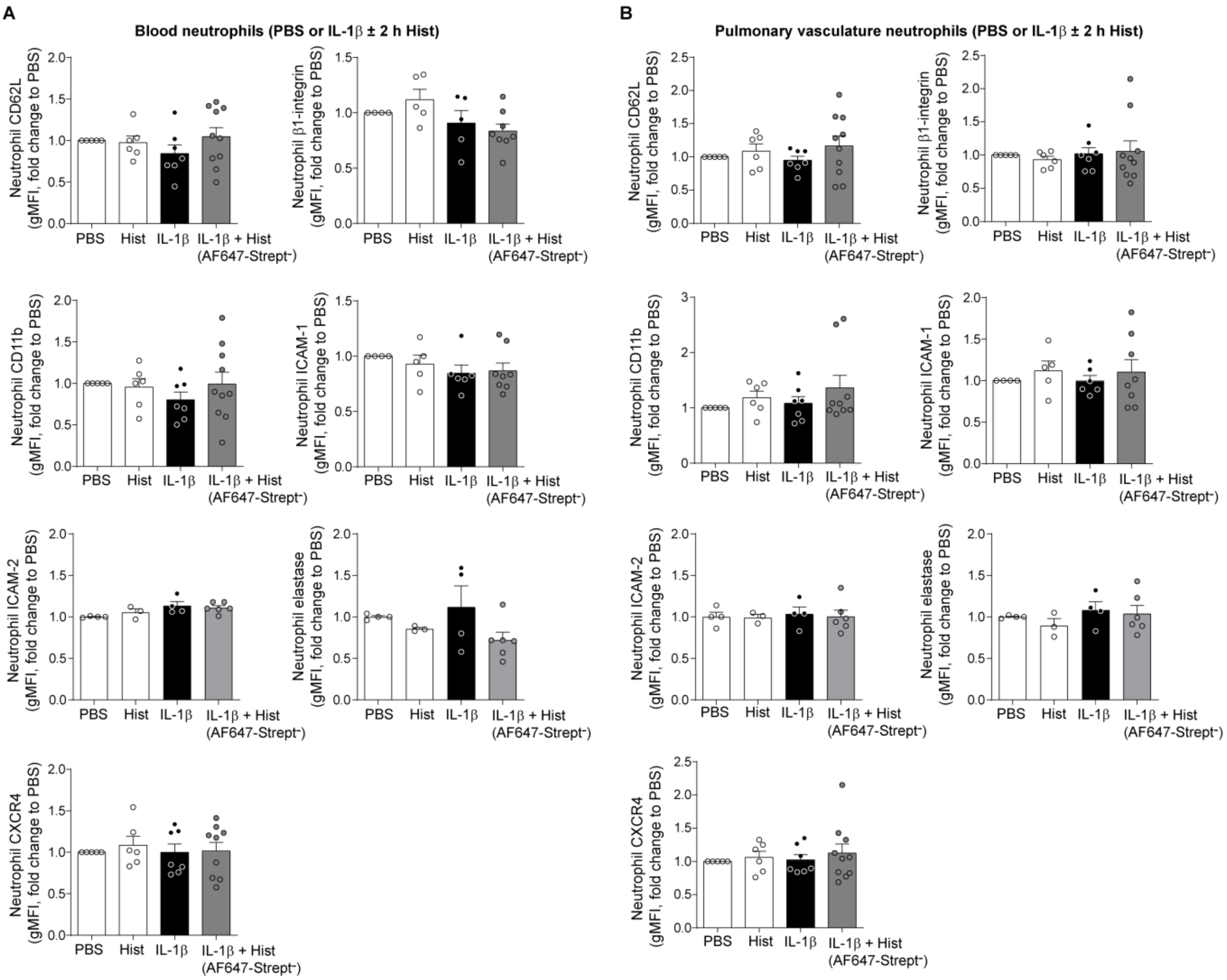

Supplemental Figure 10. Blood and pulmonary vascular washout AF647-Streptavidin$^-$ neutrophils show a phenotype similar to that of control cells. WT mice were subjected to cremaster muscle stimulation with IL-1β (50 ng) or PBS for 2 h followed by i.v. injection of biotinylated-anti-Ly6G (2 μg) at t = 90 min. The mice then received an i.s. injection of AF647-Strept (400 ng) co-administered with histamine (200 μl of 30 μM solution) or PBS for 2 hours. Peripheral blood and pulmonary vascular washout were collected and analysed by flow cytometry. (**A** and **B**) The phenotype of AF647-Streptavidin$^-$ neutrophils in blood and pulmonary vascular washout samples acquired from mice subjected to cremaster muscle stimulation with IL-1β+histamine and the biotin-Ly6G-AF647-streptavidin labelling strategy was compared to phenotype of neutrophils from unlabelled mice in which the cremaster muscles were stimulated locally with PBS, histamine and IL-1β alone. Results are presented as gMFI fold change compared to the neutrophils of samples acquired from PBS-treated mice (n = 3-10 mice per group). Data are represented as mean ± SEM (each dot represents one mouse and one independent experiment). No statistical differences were obtained between any of the groups and PBS, one-way ANOVA followed by Bonferroni's post hoc test.

References:

1. Michel CC. Starling: the formulation of his hypothesis of microvascular fluid exchange and its significance after 100 years. *Exp Physiol.* 1997;82(1):1-30.
2. Adamson RH, Lenz JF, Zhang X, Adamson GN, Weinbaum S, and Curry FE. Oncotic pressures opposing filtration across non-fenestrated rat microvessels. *J Physiol.* 2004;557(Pt 3):889-907.
3. Betteridge KB, et al. Sialic acids regulate microvessel permeability, revealed by novel in vivo studies of endothelial glycocalyx structure and function. *J Physiol.* 2017;595(15):5015-35.
4. Michel CC, Curry FE. Microvascular permeability. *Physiol Rev.* 1999;79(3):703-61.
5. Weinbaum S, Tsay R, and Curry FE. A three-dimensional junction-pore-matrix model for capillary permeability. *Microvasc Res.* 1992;44(1):85-111.
6. Kvist P, Schuster E, Lorén N, and Rasmuson A. Using fluorescent probes and FRAP to investigate macromolecule diffusion in steam-exploded wood. *Wood Sci Technol.* 2018;52(5):1395-410.
7. Li G, Yuan W, and Fu BM. A model for the blood-brain barrier permeability to water and small solutes. *J Biomech.* 2010;43(11):2133-40.
8. Armstrong JK, Wenby RB, Meiselman HJ, and Fisher TC. The hydrodynamic radii of macromolecules and their effect on red blood cell aggregation. *Biophys J.* 2004;87(6):4259-70.
9. Curry FE, Frokjaer-Jensen J. Water flow across the walls of single muscle capillaries in the frog, Rana pipiens. *J Physiol.* 1984;350:293-307.
10. Smaje L, Zweifach BW, and Intaglietta M. Micropressures and capillary filtration coefficients in single vessels of the cremaster muscle of the rat. *Microvasc Res.* 1970;2(1):96-110.
11. Arkill KP, et al. Similar endothelial glycocalyx structures in microvessels from a range of mammalian tissues: evidence for a common filtering mechanism? *Biophys J.* 2011;101(5):1046-56.
12. Squire JM, Chew M, Nneji G, Neal C, Barry J, and Michel C. Quasi-periodic substructure in the microvessel endothelial glycocalyx: a possible explanation for molecular filtering? *J Struct Biol.* 2001;136(3):239-55.
13. Huxley VH, Curry FE, and Adamson RH. Quantitative fluorescence microscopy on single capillaries: alpha-lactalbumin transport. *Am J Physiol.* 1987;252(1 Pt 2):H188-97.
14. Patlak CS, Goldstein DA, and Hoffman JF. The flow of solute and solvent across a two-membrane system. *J Theor Biol.* 1963;5(3):426-42.
15. Michel CC, Kendall S. Differing effects of histamine and serotonin on microvascular permeability in anaesthetized rats. *J Physiol.* 1997;501(Pt 3):657-62.

Supplemental movie legends

Supplemental movie 1 (related to Figure 1A). Microvascular leakage and neutrophil TEM in an IR-stimulated cremaster muscle. The movie captures a post-capillary venule after 40 min of ischemia in a *LysM-EGFP-ki* mouse. EC junctions were labelled in vivo with an AF647-anti-CD31 mAb (red). 75 kDa TRITC-dextran was injected i.v. 2 min after the beginning of the acquisition. The video shows luminal views of GFP^{bright} neutrophils (green) interacting with ECs (left panel) and trafficking of vascular dextran (blue pseudocolor intensity; right panel). The movie illustrates the development of an inflammatory response during IR with neutrophils breaching EC junctions and the occurrence of rapid and localised areas of intravascular dextran leakage (exemplified by boxed regions). Dextran signal was smoothed using a Gaussian filter for clarity using Imaris software. Still images of this video are shown in Figure 1A.

Supplemental movie 2 (related to Figure 1D). Neutrophil normal TEM in an IR-stimulated cremaster muscle. The confocal IVM movie shows a cremaster muscle post-capillary venule of a *LysM-EGFP-ki* mouse in which the TEM of a GFP^{bright} neutrophil is tracked during reperfusion phase of an IR reaction. EC junctions were labelled in vivo with an AF647-anti-CD31 mAb (red). In addition, 75 kDa TRITC-dextran was injected i.v. 2 min after the beginning of the acquisition; the signal was removed from the video for clarity. The video shows luminal views and the cross section of a selected neutrophil in high optical magnification undergoing TEM. The neutrophil was isolated from the inflammatory response for improved clarity by creating an isosurface using Imaris software. Still images of this video are shown in Figure 1D.

Supplemental movie 3 (related to Figure 1D). Neutrophil reverse TEM in an IR-stimulated cremaster muscle. The confocal IVM movie shows an IR injury response of a cremaster muscle post-capillary venule in a *LysM-EGFP-ki* mouse. The movie tracks GFP^{bright} neutrophils (green) during the reperfusion phase with EC junctions being labelled in vivo with an AF647-anti-CD31 mAb (red). The video shows luminal views and the cross section of a selected neutrophil. The neutrophil that is initially on the luminal side of the endothelium, transmigrates into the sub-EC space and subsequently migrates through EC junctions back into the vascular lumen, i.e. exhibits reverse TEM and re-enters the blood circulation. The neutrophil was isolated from the inflammatory response by creating an isosurface using Imaris software for clarity. Still images of this video are shown in Figure 1D.

Supplemental movie 4 (related to Figure 2B). Microvascular leakage and neutrophil reverse TEM as induced by IL-1β and topical histamine. The movie captures a cremaster muscle post-capillary venule in a *LysM-EGFP-ki* mouse in which GFP^{bright} neutrophils (green) are tracked post local treatment with IL-1β and topical histamine (30 μM). EC junctions were labelled in vivo with an AF647-anti-CD31 mAb (red). In addition, 75 kDa TRITC-dextran (blue pseudocolor intensity) was injected i.v. 3 min after the beginning of the image acquisition**.** The first part of the video shows the development of an inflammatory response illustrating luminal neutrophil-EC interactions. In addition, the video shows dextran extravasation after the topical application of histamine. The second part of the video shows high magnification images of a selected neutrophil within the same vessel segment undergoing reverse TEM. The neutrophil which is already in the sub-EC space migrates through EC junction in an abluminal-to-luminal direction. Dextran signal was smoothed using a Gaussian filter and the neutrophil was isolated from the inflammatory response by creating an isosurface using Imaris software for clarity. Still images of this video are shown in Figure 2B.

Supplemental movie 5 (related to Figure 4E). Interstitial 10 kDa AF488-dextran traffics through leaky venules into the blood stream. The movie shows a post-capillary venule in a WT mouse locally stimulated with IL-1β. EC junctions were labelled in vivo with an AF647-anti-CD31 mAb (blue). 75 kDa TRITC-dextran (red) was injected i.v. 2 min and 10 kDa AF488-dextran (green) was superfused 7 min after the beginning of the image acquisition. At t = 17 min, 10 kDa AF488-dextran was removed from the superfusate and replaced by Tyrode's solution containing 30 μM histamine. For clarity, EC junctions and 75 kDa TRITC-dextran signals are shown on the left side and during the first part of the video. Similarly, EC junctions and 10 kDa AF488-dextran are shown on the right side and during the second part of the video. Collectively the movie shows that immediately after topical histamine the intravascular 75 kDa TRITC-dextran rapidly leaks into the perivascular space (indicative of enhanced vascular leakage) while free flowing interstitial 10 kDa AF488-dextran disappears from the tissue compartment (or is captured by perivascular cells). Dextran signals were smoothed using a Gaussian filter for clarity using Imaris software. Still images of this video are shown in Figure 4E.

Supplemental movie 6 (related to Figure 7C). Topical AF647-streptavidin efficiently labels rTEM neutrophils. *LysM-EGFP-ki* mice were stimulated with IL-1β (50 ng for 2 h) followed by an i.v. injection of biotin-anti-Ly6G mAb (2 μg) to label blood neutrophils at t = 90 min. Tissues were then exteriorised and superfused with histamine (30 μM) in conjunction with AF647-streptavidin (1 μg/ml) for 2 h. The movie tracks GFP^{bright} neutrophils (blue) during the vascular leakage phase induced by histamine with EC junctions being labelled in vivo with an AF555-anti-CD31 mAb (green). The tracked

neutrophil that is initially on the luminal side of the endothelium, initiates TEM and during this process its leading body within the sub-EC space rapidly becomes AF647-Strept$^+$. When the neutrophil reverse migrates through EC junctions and re-enters the vascular lumen, AF647-streptavidin quickly redistributes around the surface of the cell and hence the rTEM neutrophil is effectively labelled as AF647-Strept$^+$. The neutrophil was isolated from the inflammatory response by creating an isosurface using Imaris software for clarity. Still images of this video are shown in Figure 7C.
